# Uptake of Biotin by Chlamydia Spp. through the Use of a Bacterial Transporter (BioY) and a Host-Cell Transporter (SMVT)

**DOI:** 10.1371/journal.pone.0046052

**Published:** 2012-09-27

**Authors:** Derek J. Fisher, Reinaldo E. Fernández, Nancy E. Adams, Anthony T. Maurelli

**Affiliations:** 1 Department of Microbiology, Southern Illinois University, Carbondale, Illinois, United States of America; 2 Department of Microbiology and Immunology, F. Edward Hébert School of Medicine, Uniformed Services University of the Health Sciences, Bethesda, Maryland, United States of America; University of California Merced, United States of America

## Abstract

*Chlamydia* spp. are obligate intracellular Gram-negative bacterial pathogens that cause disease in humans and animals. Minor variations in metabolic capacity between species have been causally linked to host and tissue tropisms. Analysis of the highly conserved genomes of *Chlamydia* spp. reveals divergence in the metabolism of the essential vitamin biotin with genes for either synthesis (*bioF_2ADB*) and/or transport (*bioY*). Streptavidin blotting confirmed the presence of a single biotinylated protein in *Chlamydia*. As a first step in unraveling the need for divergent biotin acquisition strategies, we examined BioY (CTL0613) from *C. trachomatis* 434/Bu which is annotated as an S component of the type II energy coupling-factor transporters (ECF). Type II ECFs are typically composed of a transport specific component (S) and a chromosomally unlinked energy module (AT). Intriguingly, *Chlamydia* lack recognizable AT modules. Using ^3^H-biotin and recombinant *E. coli* expressing CTL0613, we demonstrated that biotin was transported with high affinity (a property of Type II ECFs previously shown to require an AT module) and capacity (apparent K(m) of 3.35 nM and V(max) of 55.1 pmol×min^−1^×mg^−1^). Since *Chlamydia* reside in a host derived membrane vacuole, termed an inclusion, we also sought a mechanism for transport of biotin from the cell cytoplasm into the inclusion vacuole. Immunofluorescence microscopy revealed that the mammalian sodium multivitamin transporter (SMVT), which transports lipoic acid, biotin, and pantothenic acid into cells, localizes to the inclusion. Since *Chlamydia* also are auxotrophic for lipoic and pantothenic acids, SMVT may be subverted by *Chlamydia* to move multiple essential compounds into the inclusion where BioY and another transporter(s) would be present to facilitate transport into the bacterium. Collectively, our data validates the first BioY from a pathogenic organism and describes a two-step mechanism by which *Chlamydia* transport biotin from the host cell into the bacterial cytoplasm.

## Introduction

The *Chlamydia* are obligate intracellular Gram-negative bacterial pathogens infecting economically-important animals as well as humans. C. *pneumoniae* causes pneumonia in humans and may be a risk factor for the development of cardiovascular disease [1], [2]. *C. trachomatis* serovars A-C are responsible for trachoma, one of the leading causes of infectious blindness, while serovars D-K and LGV are the leading cause of bacterial sexually transmitted infections (STI) worldwide [3], [4]. Chlamydial STIs can result in decreased fertility in men and women as well as sterility, pelvic inflammatory disease, and life threatening ectopic pregnancies. *C. psittaci*, an avian pathogen, causes psittacosis in humans while other *Chlamydia* spp. cause disease in a variety of wild and economically important animals with the most notable being *C. abortus*, which causes infectious abortus in sheep, cattle, and goats [5].

*Chlamydia* spp. undergo a unique two-stage developmental cycle initiating with attachment of the extra-cellular form, known as an elementary body (EB), to the eukaryotic cell surface [6]. After attachment, the EB is internalized inside a host derived membrane vacuole termed an inclusion. Within the inclusion the EB differentiates into the replicative form, the reticulate body (RB), which undergoes binary fission. Approximately 18 hours post infection, the RBs differentiate asynchronously back into EBs, eventually lysing the cell after 40–72 hours (depending upon the species). During growth and development, *Chlamydia* interact extensively with the host cell using a type 3 secretion system to transport effectors into the cytoplasm leading to multiple alterations of normal host cell processes including the re-routing of lipid vesicles to the inclusion and modification of the lipid and protein content of the inclusion membrane [7], [8].

The genome sequences of eight different species and more than 30 different strains reveals that *Chlamydia* are closely related (*C. pneumoniae* and *C. trachomatis* share >80% of their proteome while *C. trachomatis* serovars D, A, and L2 share >95% of their proteome [9], [10], [11]). Despite strong genome conservation, *Chlamydia* spp. show different host and tissue tropisms and the mechanisms responsible for these differences remain unclear. *Chlamydia* spp. show multiple signs of reductive evolution typical of obligate intracellular bacteria and are auxotrophic for many essential compounds including certain nucleotides, amino acids, vitamins, and cofactors [12]. However, auxotrophic requirements, and thus metabolic capacity, vary both between species and in some cases within species [11]. In general, species previously named *Chlamydophila* (now *Chlamydia*) show slightly greater synthetic capabilities than *Chlamydia trachomatis* and *Chlamydia muridarum* with minor variations present amongst strains of a given species. While overall metabolic variation is subtle, minor variations may greatly affect infectious outcomes. The best studied example in *C. trachomatis*, *trpBA* (the tryptophan synthase genes), which are active in *C. trachomatis* genital serovars, but are present as pseudogenes in ocular serovars, allow the genital serovars to produce tryptophan from indole [13]. Functionally, this allows the genital serovars to overcome tryptophan starvation conditions brought on by IFN-γ induction of the tryptophan degrading enzyme indoleamine 2,3-dioxygenase [IDO] through the use of indole (an IDO insensitive, TrpBA substrate) supplied by the vaginal microflora. This difference in metabolism is believed, in part, to explain the ocular versus genital tissue tropism found amongst *C. trachomatis* serovars. Variations in metabolism leading to tissue tropisms also have been reported in other pathogenic bacteria and fungi, highlighting the importance of continued studies of metabolic specialization to provide insight into why highly related species infect and replicate better in some niches than others [14], [15], [16], [17], [18]. Therefore, we sought to define biotin metabolism, which differs amongst *Chlamydia* spp. to further assess the potential effects of metabolism on tropism.

**Figure 1:**
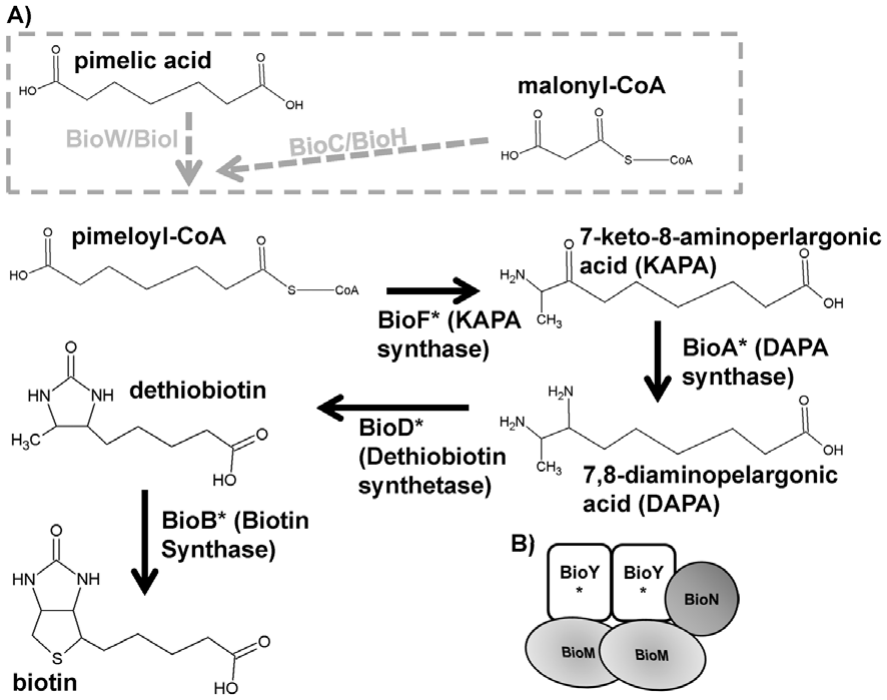
Biotin synthesis and transport in bacteria. The characterized biotin synthesis pathways (A) and the ECF biotin transport mechanism (B) are depicted. Alternative ECF subunit configurations are not shown. Asterisks indicate the presence of a chlamydial homolog based upon genome annotations. The grey dashed box indicates alternative pathways used to synthesize pimeloyl-CoA. Chemical structures were drawn using ChemSketch from ACDLabs.

Biotin, an essential vitamin for all forms of life, functions as a cofactor in carboxylation, decarboxylation, and transcarboxylation reactions by acting as a carrier of CO_2_ [19]. For biotin to be functional, it must be ligated to the ε-amino group of lysine within an apoenzyme. This process is performed by biotin ligases that in some organisms also function as regulators of genes involved in the metabolism of biotin [20]. Mammalian cells are auxotrophic for biotin and utilize two transporters for biotin uptake; the monocarboxylate transporter 1 (MCT1), which is used for biotin uptake by lymphoid cells and mitochondria, and the more widely disseminated sodium multivitamin transporter (SMVT) [21]. In contrast, many bacteria synthesize biotin from pimeloyl-CoA (which is made from different precursors) using a conserved four step process (Figure 1A) while others are auxotrophic and must acquire biotin from an exogenous source [22], [23]. Bacteria like *Escherichia coli* transport biotin using an uncharacterized mechanism while others use an energy coupling –factor transporter (ECF) [24], [25]. This recently described transporter family consists of an S component that provides substrate specificity, a membrane protein T component, and an ATPase A component [26]. For biotin transport, these genes have been designated BioY(S), BioM(A), and BioN(T) (Figure 1B). These modular units can be encoded as an operon (Type I ECFs) or as separate S components that utilize a shared AT module (type II ECFs). Many pathogenic bacteria utilize Type II ECF transporters making them attractive antibiotic targets due to the presence of a shared AT module that facilitates uptake of multiple essential compounds. Genome annotations of the pathogens *Chlamydia* and *Rickettsiales* (and other environmental bacteria) show the presence of a *bioY* in the absence of AT modules. While the BioY from *Rhodobacter capsulatus* functions independently from BioM and BioN in a recombinant *Escherichia coli* transport model, BioMN (the AT module) increases biotin transport affinity [24]. Thus it is unclear if the orphaned BioY transporters function as high or low affinity transporters or perhaps interact with highly divergent AT modules not recognized through similarity searches.

Analysis of chlamydial genomes identified differences across species (and in one case within a species) in how biotin is acquired. Some species possess the biotin synthesis genes (*bioF_2ADB*), the biotin transporter *bioY*, or both biotin synthesis and transport genes. We used recombinant *E. coli* expressing BioY (CTL0613) from *C. trachomatis* 434/Bu to determine that BioY functions as a high affinity and capacity transporter in the absence of the BioM and BioN AT module. This is the first study characterizing an orphaned type II ECF BioY and our results indicate that some S components might not require an AT module for high affinity substrate transport. Mammalian acquisition of biotin via protein transporters indicates that cell membranes are poorly permeable or impermeable to biotin. Hence, the host derived *Chlamydia* inclusion membrane also may require a biotin transporter. Immunofluorescence microscopy studies on infected cells demonstrated that SMVT colocalizes with the chlamydial inclusion membrane. These findings indicate that *Chlamydia* redirects a portion of the SMVT pool away from the cell membrane to the inclusion membrane to facilitate transport of biotin into the inclusion space where it can then be transported by BioY into the bacterium. SMVT also transports the essential compounds lipoic acid and pantothenic acid (for which *Chlamydia* are auxotrophic) into the host cell [12], [27], [28]. While previous work has shown that *Chlamydia* redirect cellular proteins to the inclusion membrane for its benefit, this is the first example to our knowledge of *Chlamydia* recruiting a cellular transporter and may serve as a paradigm for how small molecules gain access to the intra-inclusion space.

## Results

### Utilization of biotin by *Chlamydia*

*Chlamydia* encode at least two well characterized biotin utilizing proteins, the biotin carboxyl carrier protein AccB and the biotin protein ligase BirA. AccB acts in concert with AccACD to form acetyl CoA carboxylase, which converts acetyl-CoA to malonyl-CoA in the first committed step in fatty acid synthesis [29]. The biotin ligase, BirA, ligates biotin to the ε-amino group in a conserved lysine residue within AccB [30]. The chlamydial AccB proteins share 25–32% amino acid sequence identity with the *E. coli* homolog and possess an altered biotin-ligation motif sequence (consensus: AM**K**M, chlamydial: AM**K** V, Figure S1) [20]. The chlamydial BirA is a Class I biotin ligase (Class II biotin ligases contain an N-terminal helix-turn-helix motif involved in regulating the expression of biotin synthesis genes), which lacks the conserved C-terminal region found in many Class I and II BirA proteins (Figure S2) [20]. In addition to AccB and BirA, *Chlamydia spp.* encode *accACD* along with genes comprising downstream fatty acid biosynthesis pathways indicating that *Chlamydia* utilize biotin for fatty acid synthesis.

**Figure 2:**
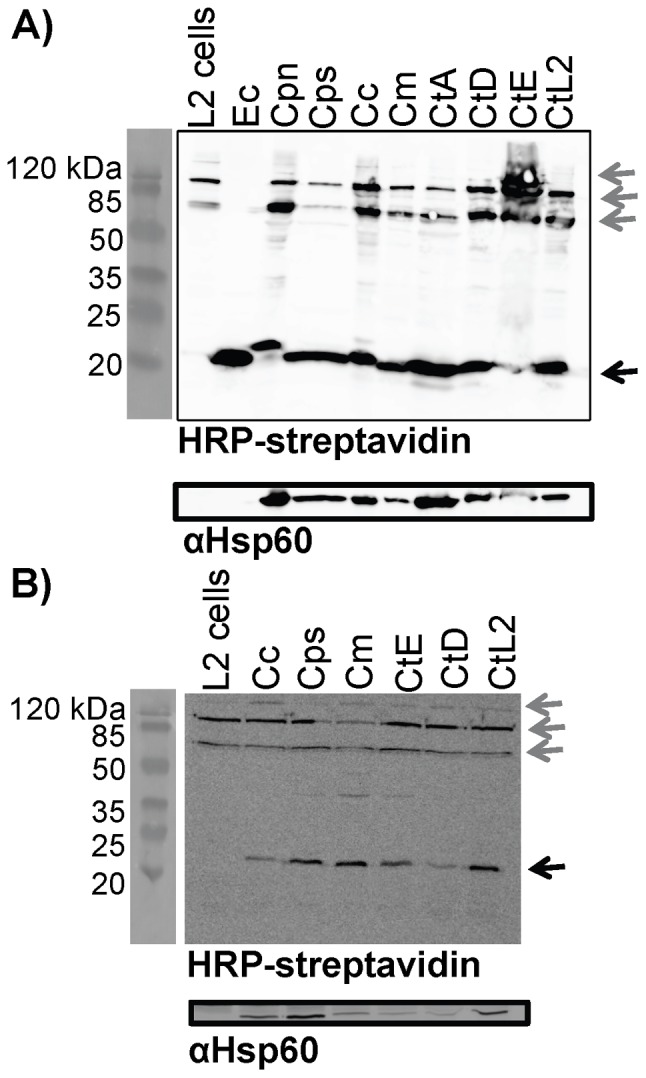
Detection of biotinylated proteins in *Chlamydia*. Protein samples were prepared from EBs (A) or L2 cells 20 hrs post infection (B) and separated on 12% SDS-PAGE for HRP-streptavidin Western blot analysis. *E. coli* lysates (Ec) and L2 cells were used as controls. The streptavidin reactive band unique to bacterial samples is indicated with a black arrow (right of blot) and prominent HRP-streptavidin reactive bands originating from host cell proteins are indicated with grey arrows (right of blot). Molecular weight markers are shown to the left of the blots. Control blots probed with anti-Hsp60 antibodies are shown beneath the HRP-streptavidin panels in panels A and B. Samples used were *C. pneumoniae* TWAR (Cpn), *C. psittaci* 6BC (Cps), *C. caviae* GPIC (Cc), *C. muridarum* NIGG (Cm), *C. trachomatis* serovar A (CtA), *C. trachomatis* serovar D (CtD), *C. trachomatis* serovar E (CtE), and *C. trachomatis* L2 strain434/Bu (CtL2).

Western blot analysis (using an HRP-streptavidin conjugate) of EB samples (Figure 2A) and cells infected for 20 hours (when RBs are the dominant developmental form) (Figure 2B) identified at least one *Chlamydia* specific biotinylated protein. *E. coli* (which contains a single covalently biotinylated protein, AccB) was used as a positive control. Samples were run under denaturing conditions resulting in the detection of only covalently modified biotinylated proteins. These blots identified a unique biotinylated protein at ∼20 kDa present in chlamydial samples that was absent in control cell lysates. The predicted mass of the chlamydial AccB is 18.2 kDa. Under our conditions, the *E. coli* AccB ran larger (∼20 kDa) than its predicted mass of 17.7 kDa. Banding patterns at the top of the blots represent biotinylated mammalian enzymes: propinoyl-CoA (60–80 kDa), methanoyl-CoA (80 kDa), pyruvate-CoA (130 kDa), and acetyl-CoA 1 and 2 (265 and 280 kDa, respectively) [31]. Control Hsp60 Western blots were run to confirm the presence of *Chlamydia* in the appropriate samples.

### Genome analysis of biotin acquisition strategies in *Chlamydia*

Analysis of the available chlamydial genomes predicts two separate pathways for biotin acquisition: synthesis starting with pimeloyl-CoA using BioF_2(BioF_1)/BioA/BioD/BioB or biotin transport using BioY (Figure 1 and Table S1). No homologs to proteins known to comprise the pimeloyl-CoA synthesis pathways in Gram-negative or Gram-positive bacteria were identified through BLAST analysis of chlamydial genomes. The chlamydial ancestor *Parachlamydia acanthamoebae* encodes proteins with weak homology to BioC (PUV_13520, 29% identity) and BioH (PUV_07140, 24% identity) while *Waddlia chondrophila* encodes a BioH homolog (wcw_1298, 26% identity). Similar to other bacteria, the biotin synthesis genes are arranged in a putative operon (*bioBF_2DA*) [32]. *Chlamydia* do not encode homologs to the known biotin synthesis gene regulators BioQ, BioR (the *C. suis* TetR protein is homologous to BioR, but likely is present due to its role in regulating tet(C) [33]), or the Class II BirA.

**Figure 3:**
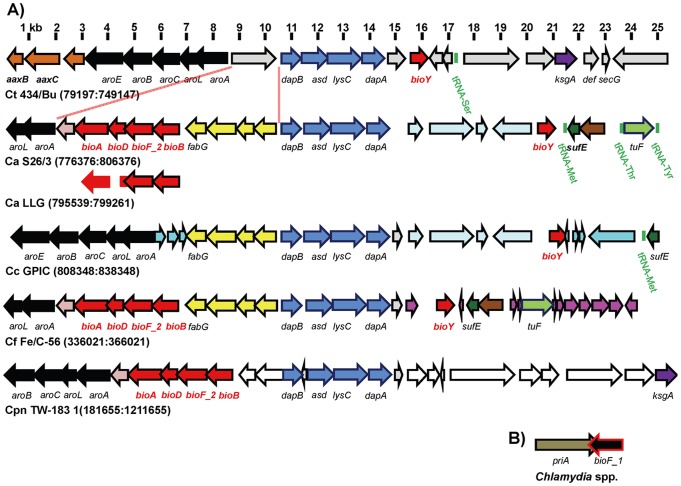
Genome synteny analysis of *Chlamydia* biotin synthesis and transport genes. Representative chlamydial genomes were anchored at the *dapB*-*dapA* gene cluster (in blue) and aligned using the Genome Region Comparison tool from JCVI CMR and NCBI genome (panel A). Genome scale is shown at the top of panel (A). Conserved gene clusters are shown in similar color. Biotin synthesis and transport genes are shown in red. In panel B, the pan-chlamydial genome location of *bioF_1* relative to *priA* is shown. The species/strains used and genome regions depicted are listed below each genome plot (Ct: *C. trachomatis*, Ca: *C. abortus*, Cc: *C. caviae*, Cf: *C. felis*, Cpn: *C. pneumoniae*). For *C. abortus* LLG, only the biotin synthesis gene operon is shown (*bioADF_2B*). The region of biotin synthesis gene insertion is shown with red lines below Ct 434/Bu.

With the exception of *C. abortus* strains S26/3 and LLG [34], variation in biotin metabolism was not observed within species (Table S1). Alignment of reference genomes anchored using the *C. trachomatis* 434/Bu *dapB/asd/lysC/dapA* gene cluster shows the localized area of genome variation accounting for divergence between biotin synthesis and/or biotin transport species (Figure 3A). A genome insertion between *aroA* and *dapB* in the group formally termed *Chlamydophila* contains the predicted biotin synthesis genes *bioBF_2DA* arranged in a putative operon (the lone exception is *C. caviae* which still contains an insertion relative to *C. trachomatis*). The genes are not arranged in the order of biotin synthesis and, consistent with the absence of known biotin gene regulators in *Chlamydia*, biotin regulator binding sites are not present upstream of the *bioB* initiation codon.

*Chlamydia* possess two *bioF* homologs, *bioF_1 and bioF_2*, which are predicted to encode a 7-keto-8-aminoperlargonate (KAPA) synthase. BioF carries out the first committed step in biotin synthesis converting pimeloyl-CoA to KAPA (Figure 1A). The *bioF_2* gene is encoded as part of the biotin synthesis gene cluster and BioF_2 is more closely related to the characterized BioF from *E. coli* than BioF_1 (Figure S3). The *bioF_1* gene is encoded separately from the biotin synthesis/biotin transport gene region where it converges upon and overlaps with the 3′ end of *priA* (Figure 3B). While both BioF_1 and BioF_2 contain a pyridoxal phosphate-dependent aspartate aminotransferase fold, BioF_1 lacks residues important for activity of the *E. coli* BioF including a stretch of 21 N-terminal amino acids (Figure S3) [35]. Based on genome annotations, the *bioF_1* gene in *C. pneumoniae*, *C. pecorum*, *C. trachomatis*, and *C. muridarum* also encode an alternative initiation codon, TTG, which was verified using Sanger sequencing of the *bioF_1* region in *C. pneumoniae* TW-183 and *C. trachomatis* 434/Bu. Consistent with these sequence differences, neighbor joining analysis separates BioF_1 and BioF_2 (clustering BioF_2 closer to BioF from *E. coli*) and distinguishes between BioF_1 with and without an ATG start codon (Figure S3B).

The *bioY* gene is located approximately one to six kb downstream from *dapA* in all strains (except *C. pneumoniae* which is *bioY*-negative) and displays ∼52% amino acid identity (42%–85%) with respect to other *Chlamydia* and ∼19% amino acid identity (14%–24%) with the previously characterized Class I ECF BioY transporters (Figure S4) [24], [36], [37], [38]. The absence of the biotin energy module genes BioM and BioN adjacent to the chlamydial *bioY* leads to a Class II ECF designation and would predict that an AT module should be encoded elsewhere to be shared by solute-specific S transporter proteins [26]. However, unlike standard type II ECFs, *Chlamydia* encode no identifiable AT gene homologs.

### Biotin transport across the bacterial membrane

To characterize the transport properties of BioY, we constructed a recombinant N-terminal His-tagged version of CTL0613 from *C. trachomatis* 434/Bu for expression in *E. coli* (*E. coli* ATM1172). The obligate intracellular life-style of *Chlamydia* and the absence of characterized gene expression systems often necessitate the use of an alternative bacterial host for the study of chlamydial proteins. C43(DE3), the *E. coli* host strain used for BioY expression, is derived from *E. coli* BL21(DE3) [39]. Consistent with a previous study that rigorously analyzed bacteria for the presence of ECFs, our analysis of the BL21(DE3) genome revealed no ECF AT modules that would potentially interfere with our analysis of the orphaned chlamydial BioY [26], [40]. Anti-His-tag Western blotting demonstrated that BioY was expressed by *E. coli* ATM1172 and migrated primarily at a calculated mass of 37 kDa with a minor band at 21 kDa (Figure S5). The expected mass of a His-tagged BioY monomer and dimer are 24 and 48 kDa, respectively.

**Figure 4:**
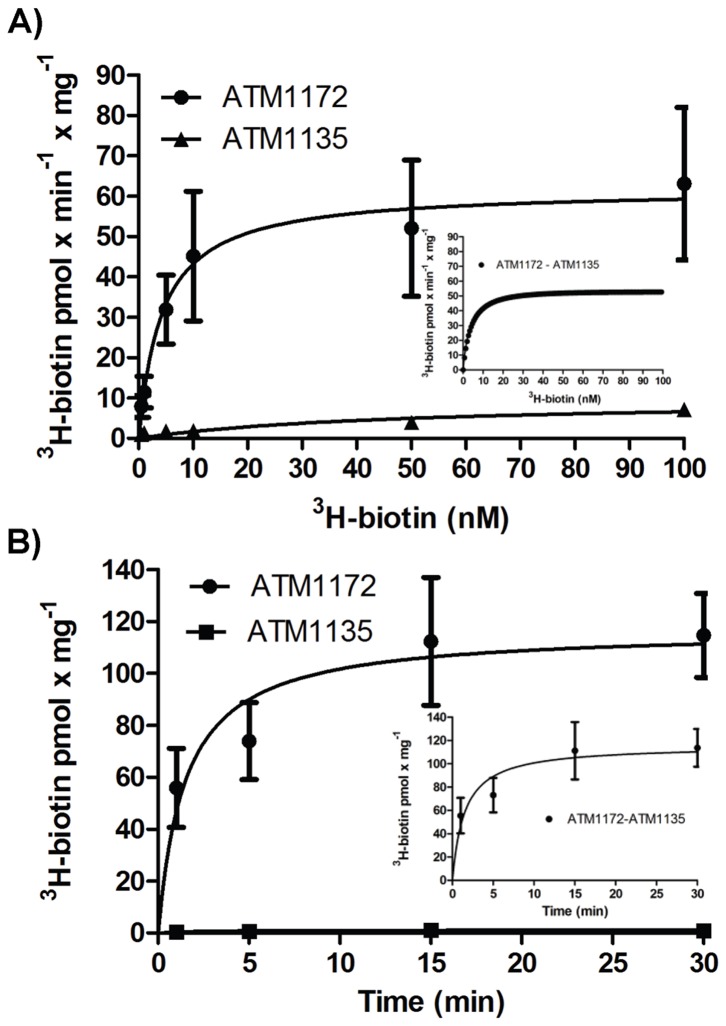
Transport of ^3^H-biotin in *E. coli* mediated by *Chlamydia* BioY. BioY transport kinetics were determined in (A) by incubating *E. coli* ATM1172 and *E. coli* ATM1135 with 0.5, 1, 5, 10, 50, or 100 nM ^3^H-biotin for one minute at 30°C. Reactions were halted and the amount of radioactivity present in the sample was measured using a scintillation counter. Data were plotted and analyzed using GraphPad 5.0. In (B), *E. coli* ATM1172 and *E. coli* ATM1135 were incubated with 100 nM ^3^H-biotin at 30°C for 1, 5, 15, or 30 minutes. The inserts in (A) and (B) show corrected values after subtracting *E. coli* ATM1135 background levels.

**Figure 5:**
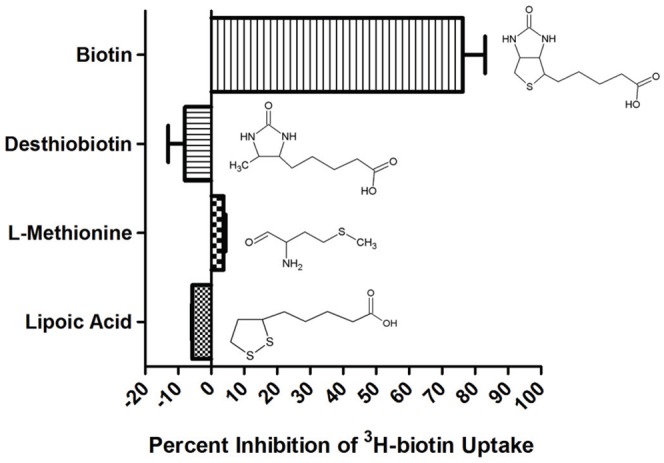
Specificity of transport for ^3^H-biotin. Bacterial samples were incubated with 10 nM ^3^H-biotin and 1 µM of competitor at 30°C for 1 minute. Reactions were then halted and the amount of radioactivity in the samples was determined. Values are reported as the percent inhibition of ^3^H-biotin uptake compared to a no inhibitor control.

We next analyzed the transport properties of BioY using a ^3^H-biotin uptake assay. Preliminary experiments demonstrated that BioY transport properties were not adversely affected by the presence of the N-terminal His-tag (Figures S6 and S7) and that uptake of biotin by *E. coli* ATM1172 was significantly higher than *E. coli* ATM1135, the host strain carrying the empty vector (Figure S6). *E. coli* ATM1172 transported biotin with an apparent K(m) of 3.35 nM (95% confidence interval 3.27 to 3.42) and an apparent V(max) of 55.1 pmol×min^−1^×mg^−1^ (95% confidence interval of 55.0 to 55.3) (Figure 4A). Consistent with carrier mediated transport, ^3^H-biotin transport was linear over the first minute of incubation and reached saturation within approximately 10 minutes (Figure 4B). The transport specificity of BioY was analyzed by measuring the inhibition of ^3^H-biotin transport in the presence of biotin, the sulfur containing compounds lipoic acid and L-methionine, or the biotin precursor desthiobiotin. Transport of ^3^H-biotin was significantly inhibited only in the presence of biotin (Figure 5).

### Transport of biotin across the inclusion membrane

**Figure 6:**
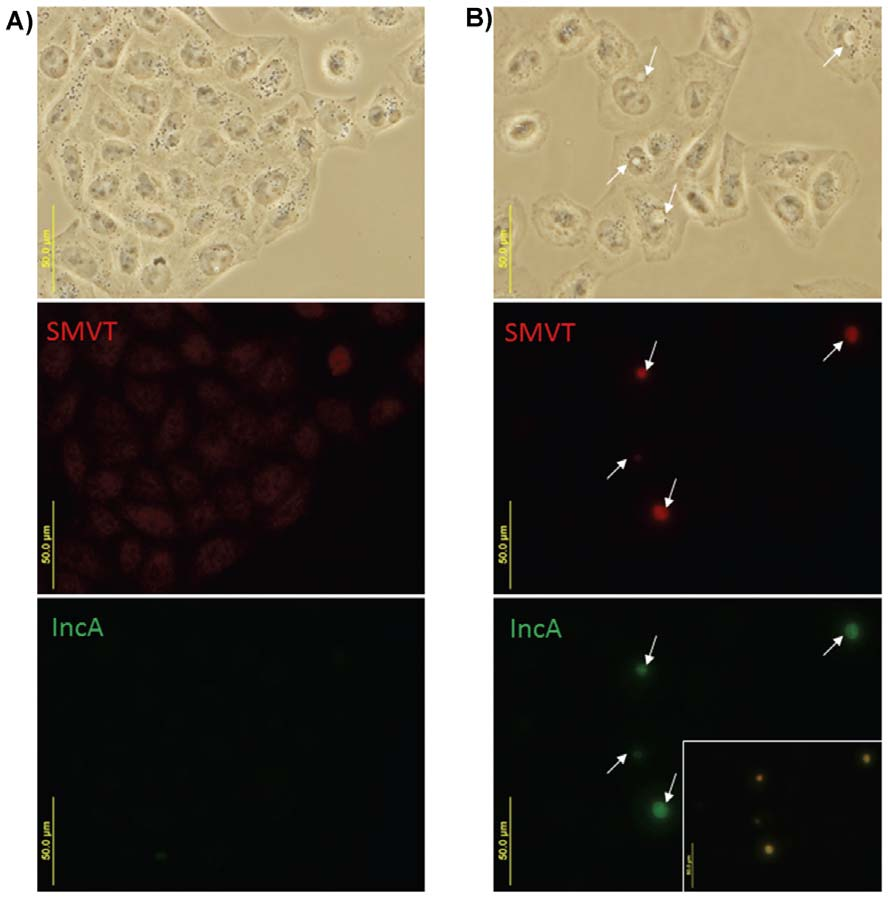
Localization of SMVT in infected HeLa cells using immunofluorescence microscopy. HeLa cells were mock infected (A) or infected with *C. trachomatis* 434/Bu (B) for 20–24 hours, fixed and permeablized, and probed with anti-IncA antibodies (shown in green) and anti-SMVT (H56) antibodies (shown in red). The location of inclusions viewed under phase contrast (top panel) and fluorescence (middle and bottom panels) are shown with white arrows. The inset in panel (B) was created by merging the red and green channel micrographs.

Since the membrane of the *Chlamydia* inclusion is derived from the host cell which is poorly permeable of impermeable to biotin [19] and previous studies were unable to measure inclusion permeability to molecules between 45 and 520 Da (biotin has a mass of 244 Da), we hypothesized that the inclusion also requires a transporter to import biotin [41], [42]. SMVT localization was initially studied using antibodies that recognize the C-terminus of SMVT (H56) in both infected and mock-infected HeLa cells. Antibodies against the *C. trachomatis* inclusion protein IncA were used to define the inclusion. In uninfected cells, SMVT had a diffuse, punctate staining pattern across the cell surface (Figure 6A). In contrast, infected cells had an intense SMVT signal that co-localized with the IncA antibody staining pattern (Figure 6B). To confirm these findings, experiments also were performed using an antibody raised against the N-terminus of SMVT (N14). While detection of SMVT with N14 in uninfected cells was less obvious than with H56, colocalization with IncA staining was easily visualized (Figure S8A and B). Similar to experiments with *C. trachomatis* 434/Bu, SMVT (detected using the H56 antibody) also localized to the chlamydial inclusion in HeLa cells infected with *C. trachomatis* E, *C. psittaci* 6BC, and *C. caviae* GPIC (Figure S8C). Both *C. trachomatis* E and *C. caviae* GPIC are biotin auxotrophs while *C. psittaci* 6BC possesses genes for biotin synthesis and transport (Table S1). To demonstrate that these results were not a HeLa specific phenomenon, we also tested for colocalization of SMVT with *C. trachomatis* 434/Bu inclusions (using anti-SMVT H56 and anti-IncA antibodies) in infected McCoy mouse cells and obtained identical results (Figure S9).

## Discussion

Obligate intracellular bacteria exist under a unique “living arrangement” enabling them to entirely or partially shed energetically costly metabolic pathways if they possess mechanisms to parasitize the host for the essential intermediate or end products. However, the ensuing evolutionary reduction in metabolic capacity and subsequent reliance on the host cell may result in host or tissue tropisms due to growth restrictions arising from differences in cellular nutrient content and bioavailability. Thus, bacteria risk becoming overly specialized as a consequence of their diminished metabolic versatility. Elucidation of the underlying genotypic and phenotypic reasons for tropisms has the potential to illuminate an Achilles heel to be targeted by chemotherapeutics or vaccines. Of course, a pathogen's metabolic repertoire will not be the sole factor(s) driving tropism, but will function in concert with more traditional virulence factors.

Despite their close genomic relationship *Chlamydia* spp. have different host and tissue tropisms. The previously classified *Chlamydophila* family members, with the exception of *C. caviae*, generally have a greater host range and can propagate in more tissue types than *C. trachomatis* and *C. muridarum* [43]. Genome sequences for all species except *C. suis* have now been published and genome comparisons reveal that divergence primarily lies within the plasticity zone (which includes two toxin-like genes along with tryptophan synthesis and purine/pyrimidine salvage pathways), the type V transporter repertoire (Pmps), putative T3SS effectors (which includes the Inc family proteins), and metabolism [11]. Analysis of metabolic capacity of *Chlamydia* spp. based upon genome annotations shows differing abilities to salvage nucleotides, synthesize tryptophan, transport pantothenic acid, degrade arginine, and acquire biotin. However, functional analysis of AaxB and AaxC from *C. trachomatis* serovars determined that missense mutations led to inactive proteins [44]. Loss of activity was not predicted based upon sequence analysis alone, hinting that additional pseudogenes may be present and that metabolic divergence may be greater than appreciated.

Biotin has long been known to be an essential vitamin for mammals with biotin deficiencies and mutations in biotin utilizing enzymes resulting in multiple defects apparent at both the cellular and organismal level [45]. Mammals primarily acquire biotin from dietary sources via carrier mediated uptake. The primary biotin uptake system is the SMVT, a 12 transmembrane protein that is widely expressed throughout the body [46]. SMVT also facilities the acquisition of two other essential compounds, pantothenic acid and lipoic acid. At least one other transporter, MCT1, is known to be utilized for uptake of biotin by lymphoid cells and potentially mitochondria [19]. Once inside the cell, biotin is covalently attached to the ε-amino group of a conserved lysine within apoenzymes by a biotin protein ligase. Mammalian cells utilize five biotin requiring enzymes for various trans-, de-, and carboxylation reactions in which biotin serves as a CO_2_ carrier.

Bacteria generally encode fewer biotin requiring enzymes than mammalian cells, with *E. coli* known to contain only one covalently modified enzyme (AccB) along with the biotin protein ligase BirA to which biotin is bound non-covalently as AMP-biotin [30]. Similar to *E. coli*, *Chlamydia* only contain one annotated biotinylated protein, AccB, and a single biotin protein ligase, BirA. HRP-streptavidin blotting analysis of both the EB and RB developmental forms revealed a single band that was absent in protein samples from uninfected cells. This band size is larger than the expected size of the chlamydial AccB (>20 kDa versus 18.2 kDa). However, similar results were seen for the *E. coli* AccB (>20 kDa versus 17.7 kDa) and are consistent with other studies [47], [48]. Thus, it is highly likely that the single reactive band from *Chlamydia* is AccB. Higher weight reactive bands seen in all EB, RB, and uninfected cell samples correspond to the predicted sizes of the mammalian biotin-utilizing enzymes. Contamination of EB preparations with host proteins is common and RB samples were prepared from lysates of infected HeLa cells [49]. The bands migrating between AccB and the higher weight bands (>50 kDa) could be AccB multimers or biotinylated histones [50]. Since biotin is not covalently attached to BirA we did not expect to see a BirA band under denaturing gel conditions. Proteomics studies have identified all of the acetyl-CoA carboxylase complex components (ACC, comprised of AccABCD) in *C. trachomatis* EBs and RBs, AccBA in *C. pneumoniae* EBs, and lipid analysis has demonstrated that *Chlamydia* synthesize fatty acids *de novo* [49], [51], [52], [53]. Our data further support the role of the ACC and suggest that the holoenzyme may be present in EBs (and, not surprisingly, RBs) indicating that fatty acid synthesis may be required during immediate early infection. If this model is correct, then ACC would make a prime target for antimicrobials.

BirA from *Chlamydia* is a Class I biotin protein ligase (BPL). Class I BPLs lack the N-terminal helix-turn-helix DNA binding motif (utilized for regulation of biotin synthesis genes) that defines the Class II BPLs [20]. The chlamydial BirA also lack a conserved C-terminal region of unknown function found in most Class I and II BPLs. The likely presence of a biotinylated AccB-like protein in *Chlamydia* demonstrates that BirA is functional and transcriptome data indicate that BirA is present throughout development [54], [55]. The Class I BPL in *Chlamydia* and the absence of homologs to other known biotin gene regulators [32] suggests that either the biotin synthesis/transport genes do not need to be regulated or that regulation occurs through a novel mechanism. Species with a single biotin acquisition strategy (BioY or synthesis) may not require regulation. However, for species possessing both *bioY* and synthesis genes a lack of regulation would result in the use of redundant pathways. As biotin synthesis is a costly metabolic pathway, biotin transport via BioY would seem like the preferred pathway except when biotin is limiting [22]. Analysis of biotin tissue content in rats and chickens found that biotin levels are low in lung and heart tissue compared to other organs [56]. This could explain why *C. pneumoniae*, which primarily infects the lungs, has dispensed with *bioY*. Furthermore, species with greater host and tissue range (*C. psittaci, C. abortus*, and *C. felis*) maintain both biotin synthesis and transport genes which may allow them to switch between energy intensive synthesis versus low-energy transport depending on biotin availability at the site of infection. The recently reported sequence of *C. abortus* LLG was found to have a putative degraded biotin synthesis pathway (Table S1) making it the only example of biotin metabolism variation within a species [34]. *C. abortus* can grow in a number of hosts and tissues, but is best known for causing abortions. Pregnant animals are known to incur transient biotin deficiencies and mouse models have shown that reduced biotin levels in dams correlates with reduced levels of biotin in the fetus [57], [58]. The loss of biotin synthesis by the LLG strain might explain its reduced pathology in a pregnant mouse abortion model and small plaque phenotype and makes it an ideal candidate to test for biotin dependent phenotypes compared to “dual” biotin metabolism *C. abortus* strains [59].

The biotin synthesis *bioBF_2DA* genes in *Chlamydia* would facilitate biotin synthesis starting from pimeloyl-CoA. Global expression analysis indicates that these genes are expressed in *C. pneumoniae* showing maximum levels during the mid-cycle when bacterial division and fatty-acid synthesis would be maximal [55]. In Gram-negative bacteria, pimeloyl-CoA is synthesized by BioC and BioH (Figure 1) [22]. *Chlamydia* lack homologs to BioC/BioH, but do possess a FabH which shares 33% identity with BioZ (a FabH-like protein) from *Mezorhizobium sp.* strain R7A. In *M*. RZA BioZ is predicted to function in both fatty acid synthesis (as FabH) and biotin synthesis as BioH due to relaxed substrate specificity compared to the *E. coli* FabH [60]. A similar mechanism would not be surprising for *Chlamydia* spp. which are known to encode other rare or novel bifunctional enzymes [61], [62]. It is unclear if *Chlamydia* encode a BioC homolog but the best candidate based on similarity is UbiE, which shares a SAM dependent methyltransferase domain with BioC.

Annotations of the biotin synthesis pathway in *Chlamydia* list two *bioF* genes, *bioF_1* (located next to *priA*) and *bioF_2* (encoded within the biotin synthesis operon), which share an aspartate aminotransferase fold type I. BioF_2 shows greater similarity to the characterized synthetic BioF proteins, shares more active site residues with BioF, and is present with the other biotin genes making it likely to be the bona fide BioF. We predict that BioF_1 participates in an amino-transferase reaction separate from biotin synthesis, consistent with its maintenance and expression in biotin synthesis deficient species, and should be reclassified as a conserved hypothetical protein until functional analysis can assign a proper role.

Biotin transport in bacteria has been recognized for over 35 years [25], [63], however the actual transport proteins have only recently been determined and still remain elusive for *E. coli*. The ECF transport family described by Rodoniov *et al.* comprises a collection of small molecule transporters with diverse substrate preference, but conserved membrane energy coupling components [26]. Studies with the *Rhodobacter capsulatus* BioYMN have demonstrated that the S component can function independently as a low affinity, high capacity transporter, but is converted to a high affinity, low capacity transporter in the presence of the BioMN module [24]. The Class I BioYMN from other bacteria also function similar to the BIOYMN from *R. capsulatus* (Table S2). Whether the non-*R. capsulatus* Class I BioY transporters show altered transport in the absence of BioMN is unknown. Genome analysis by Rodionov *et al.* for ECF transporters identified BioY as the only S component to be encoded by bacteria (including the *Rickettsiales* and *Chlamydia*) in the absence identifiable AT modules suggesting that BioY may function at a physiologically relevant level in its orphan form which may be a unique property of BioY [26], [64].

Surprisingly, in our heterologous expression system BioY showed high affinity and moderate capacity transport in the absence of BioMN (bioinformatic analysis did not identify AT modules in *E. coli* BL21(DE3)). Previous heterologous studies of BioYMN used an *E. coli* K12 derivative S1039 due to its absence of the *E. coli* high affinity biotin transport [24], [40]. However, the high affinity biotin transport system in *E. coli* has not been defined and the mechanism underlying its absence in S1039 is not understood. Our preliminary experiments indicated that expression of BioY in the BL21(DE3) background significantly increased transport above background levels (Figure S6) and the apparent K(m) and V(max) were both superior to those determined for *E. coli* ATM1135 (Table S2). The ability of BioY to function as a high affinity transporter in the absence of BioMN may have made BioMN dispensable, leading to loss of the AT module in a subset of bacteria including *Chlamydia*. Berntsson *et al.* argue in a recent report that BioY from *Lactococcus lactis* (a Class II transporter) does not function as a transporter in the absence of a shared MN module, but merely binds biotin [65]. However, the absence of identifiable MN-encoding genes in *Chlamydia* and the differences in BioY amino acid sequences between these species (<25%) suggests that the chlamydial BioY may have altered functionality. Amino acid sequence analysis does not reveal obvious reasons for these transport differences and the regions of BioY that interact with BioN or BioM are not known. Work focusing on the stoichiometry of ECF components predict an SA_2_T for non-BioY ECFs while the BioYMN system is predicted to exist in an S_2_A_2_T ratio or possibly as an S_2_A_2_T_2_ [40], [64], [66]. Consistent with this observation our recombinant BioY ran primarily as a dimer (Figure S5). Interestingly, BioY is present in many bacteria in two copies (including the *Chlamydia* relative *Simkania*) [26] suggesting that perhaps a low affinity complex (requiring BioMN) and high affinity complex could be assembled with the loss of the low affinity complex in some species accompanied with the loss of the AT module. Further biotin transport studies using Class I and Class II BioY transporters will be required to address this point including comparisons of the transport affinities of BioY from species encoding two copies of *bioY* with and without a heterologous or homologous BioMN as well as studies to differentiate binding versus transport in these systems.

The dependence on exogenous biotin by some *Chlamydia* species presents a unique problem, namely, how does biotin get across the inclusion membrane? Studies have determined that the inclusion membrane is permeable to small ions, but is impermeable to polymers larger than 520 Da leaving a window of 45–520 Da in which permeability is unclear [41], [42]. During infection *Chlamydia* maintain intimate interactions with the host cell altering host cell immune function, organelle architecture, and vesicle trafficking patterns [67], [68], [69]. Using the microtubule network the inclusion positions itself within the exocytic pathway, avoiding lysosomal fusion, while at the same time situating itself to intercept various vesicles and proteins [70]. These exocytic vesicles either fuse with the growing inclusion membrane or are taken up into the inclusion space where they interact directly with RBs [71], [72]. *Chlamydia* can also retarget host proteins normally found in the cytosol or ER to the inclusion membrane [73], [74]. While vesicles could potentially delivery some molecules to the inclusion space upon fusion with the inclusion membrane, not all compounds for which *Chlamydia* are auxotrophic would be contained in vesicles.

The redirection of vesicles and proteins to the inclusion membrane led us to seek a host cell protein to solve *Chlamydia*'s biotin “dilemma”. SMVT is an ideal candidate for a number of reasons including: 1) its ability to transport of biotin, lipoic acid, and pantothenic acid for which *Chlamydia* are auxotrophic, 2) its presence in tissues infected by *Chlamydia*, and 3) its reliance for transport on microtubules and dynein, cellular components that *Chlamydia* are known to modulate [45], [70], [75]. Immunofluorescence microscopy demonstrated co-localization of SMVT with the inclusion membrane in two different host cells using biotin auxotroph species and a facultative biotin prototroph. The K(m) of SMVT for biotin was determined to be 15.1 µM [27], which is significantly higher than for BioY (3.35 nM) and should allow *Chlamydia* to establish influx of biotin from the host cell into the bacterium. While not tested, we predict that SMVT also would co-localize to the *C. pneumoniae* inclusion despite its biotin prototrophy due to its need for exogenous lipoic acid and pantothenic acid. It should be noted that the requirement for exogenous pantothenic acid is less certain than for lipoic acid [28] due to the absence of annotated genes to carry out the conversion of pantothenic acid to its functional form, CoEnzyme A. How lipoic and pantothenic acid would then be transported into the bacterium is unclear. The formerly classified *Chlamydophila* species possess a PanF homolog, which functions as a Na/pantothenic acid symporter in *E. coli* while all *Chlamydia* lack homologs to known lipoic acid transporters.

The co-localization of SMVT with the inclusion membrane is consistent with, but does not prove, that it is inserted into and utilized by the inclusion to transport biotin and other vitamins across the inclusion membrane. Use of SMVT would further support the sensitivity of *Chlamydia* to microtubule disrupting agents as these compounds would not only affect lipid acquisition, but also would result in the loss of nutrients due to the failure of SMVT to route to the inclusion. The use of SMVT would set a precedent for the requirement of transporters to move compounds between 45 and 520 Da, such as nucleotides, amino acids, and SAM (for which bacterial transporters have been characterized) into the inclusion. Recent work from Saka *et al.* identified Npt1, the chlamydial ATP/ADP translocase, in inclusion membrane fractions as well as in RB fractions providing another mechanism to circumvent the inclusion permeability problem [51]. Coupled with our findings, it seems that *Chlamydia* may utilize both bacterial and host transporters to acquire compounds from the host cytosol. It will be interesting to determine if other chlamydial transporters also reside in the inclusion and why some transporters can function as bacterial and inclusion transporters while others only function in one locale. Together these data expand upon our understanding of basic chlamydial biology, further defining its metabolic requirements and ability to modulate host cell factors to its advantage.

## Materials and Methods

### Cell culture conditions

Cell lines (listed in Table S3) were routinely cultured in T-175 culture flasks (BD Falcon) using Dulbecco's Modified Eagle Medium+GlutaMAX™ [Gibco®] (DMEM) supplemented with 10% heat-inactivated Fetal Bovine Serum (FBS, Hyclone) at 37°C with 5% CO_2_. During infection experiments, cell medium was supplemented with 1× MEM Non-Essential Amino Acids Solution (Sigma) and 0.2 µg/ml cyclohexamide (Sigma). Cells were routinely monitored for *Mycoplasma* infection by PCR using DNA lysates (QIAgen DNeasy Kit) with degenerate oligos that prime to the 16S rRNA of *Mycoplasma* spp.

### Cell infection and bacterial growth conditions

The bacterial strains used in this study are listed in Table S3. EBs were harvested from either L2 cells or Hep2 cells (for *C. pneumoniae*) and stored at −80°C in sucrose phosphate glutamic acid buffer [7.5% W/V sucrose, 17 mM Na_2_HPO_4_, 3 mM NaH_2_PO_4_, 5 mM L-glutamic acid, pH 7.4] until use. Stocks were titered using an infection forming unit assay (IFU) and tested for *Mycoplasma* contamination using PCR. *E. coli* were routinely cultured in Luria Bertani medium at 37°C with 100 µg/ml ampicillin (Sigma) as appropriate.

### Construction of *E. coli* ATM1172 and *E. coli* ATM1173

Genomic DNA was isolated from *C. trachomatis* 434/Bu EBs using the QIAgen DNeasy Kit. Primers Y+His5 [5′-AAGCATATGGTTGCTAA GAGCATTACTAAAG-3′] and Y19b3 [5′-GATCCTCGAGTTATTTTCTGATAAATAATCTT C-3′] were used to clone CTL0613 in frame with the N-terminal His-tag sequence in pET19b (EMD chemicals). Primers Y-His5 [5′- ATACCATGGTTGCTAAGAGCATTAC-3′] and Y19b3 were used to create a tagless CTL0613 construct still expressed from the T7 promoter in pET19b. Restriction sites are underlined. Both sequences were PCR amplified using Platinum Taq DNA polymerase (Life Technologies) as directed. PCR products were subcloned into pGEM-T (Promega), digested using either NdeI and XhoI (for cloning with the His-tag) or NcoI and XhoI (for cloning without the His-tag), and ligated into similarly digested pET19b. Final vectors were sequence verified using T7 promoter (5′- TAATACGACTCACTATAGGG-3′) and T7 terminator (5′- GCTAGTTATTGCTCAGCGG-3′) primers at the Uniformed Services University Biomedical Instrumentation Center (Bethesda, MD) and transformed into *E. coli* C43(DE3) (Lucigen) to create *E. coli* ATM1172 and *E. coli* ATM1173. C43(DE3) carrying pET19b (*E. coli* ATM1135) also was constructed for control experiments.

### Western blot detection of proteins

#### Detection of biotinylated proteins

To detect biotinylated proteins from mid-developmental cycle *Chlamydia*, confluent L2 cell monolayers grown in 60 mm tissue culture dishes were infected with EBs at an MOI of 1 (or mock infected) as previously described [44]. At 20 hours post infection, the medium was removed and cells were washed with PBS. Cells were then lysed by the addition of 200 µl of Laemmli buffer containing 25 units benzonase (EMD) and 1× Halt Protease Inhibitor Cocktail (Thermo Scientific). After incubating for five minutes on ice, samples were collected and β-mercaptoethanol was added (358 mM final) prior to boiling. For the detection of biotinylated proteins in EBs, EBs were lysed in Laemmli buffer containing 1× Halt Protease Inhibitor Cocktail and β-mercaptoethanol (358 mM final). *E. coli* control samples were obtained from cultures grown in Luria Bertani broth, which were harvested by centrifugation and lysed with Laemmli buffer containing 1× Halt Protease Inhibitor Cocktail and β-mercaptoethanol. Samples were run on 12% SDS-PAGE gels (10 µl of cell lysate, 2×10^6^ EBs, or 2×10^6^ *E. coli* C43(DE3) (Lucigen)) and then transferred to nitrocellulose. Blots were blocked with 1% BSA/PBS, washed with 0.05% Tween 20/PBS and then incubated with Ultrasensitive Streptavidin-Peroxidase Polymer (Sigma) diluted 1∶1000 in 1% BSA/0.05% Tween 20/PBS. After probing for biotinylated proteins, blots were washed with 0.05% Tween 20/PBS, incubated with Supersignal West Pico Chemiluminescent Substrate (Thermo Scientific) and visualized using an ImageQuant LAS 4000 (GE Healthcare).

#### Detection of Hsp60 and His-tagged BioY

Samples prepared for biotinylated protein analysis also were used for Hsp60 Western blot analysis. After transferring proteins to nitrocellulose, blots were blocked with 5% milk/TBS and probed with a mouse anti-Hsp60 antibody (A57-B9, provided by Dr. Dan Rockey, Oregon State University) diluted 1∶1000 in 5% milk/0.05%Tween-20/TBS. Blots were then washed with 0.05%Tween-20/TBS, probed with a HRP-conjugated goat anti-mouse antibody (GE Healthcare), and finally washed again in 0.05%Tween-20/TBS. To detect His-tagged BioY, *E. coli* ATM1172 was grown in 2xTY medium (8 g tryptone, 5 g yeast extract, and 5 g NaCl per liter adjusted to pH 7.0) with 100 µg/ml ampicillin (Sigma) to an OD_600 nm_ of ∼0.6 and then induced with 1 mM IPTG (Sigma) at 37°C for 2.5 hours. Bacteria were then pelleted and lysed in Laemmli buffer containing 1× Halt Protease Inhibitor Cocktail and β-mercaptoethanol, boiled, and run on 12% SDS-PAGE gels. Protein was transferred to nitrocellulose and blotted as described for Hsp60 with the exception that a mouse anti-His tag antibody (GE Healthcare) diluted 1∶1000 was substituted for the anti-Hsp60 antibody. Blots were developed using Supersignal West Pico Chemiluminescent Substrate and visualized using an ImageQuant LAS 4000.

### ^3^H-biotin transport assays

To measure biotin transport, *E. coli* ATM1172, *E. coli* ATM1173, or *E. coli* ATM1135 were grown overnight in 2xTY medium with 100 µg/ml ampicillin at 37°C. Bacteria were then subcultured into 2xTY containing 100 µg/ml ampicillin and 0.1 mM IPTG and grown at 37°C to an OD_600 nm_ of ∼1. Cultures were placed on ice and then pelleted to concentrate samples. The pellets were washed twice with ice cold M9 minimal salts and finally suspended in M9 minimal salts to ∼4×10^9^ bacteria per ml. Experiments were performed with 50 µl samples containing ∼2×10^8^ bacteria and ∼0.1 to 0.5 mg/ml protein. Protein levels were quantified using the Bio-Rad Protein Assay. For ^3^H-biotin uptake experiments, bacteria were mixed with 50 µl of D-[8,9-^3^H(N)] biotin (American Radiolabeled Chemicals, Inc) and incubated at 30°C for the desired time interval (see Results section for time periods and amounts of ^3^H-biotin utilized). Reactions were stopped by adding the reaction mixture to 20 ml ice cold PBS, filtering through a 0.45 µm Durapore membrane filter, and washing once with 20 ml ice cold PBS. The filters were then placed into scintillation vials with 5 ml ReadySafe liquid scintillation cocktail (Beckman Coulter) and disintegrations-per-minute were recorded. Radioactivity was normalized by converting the disintegrations-per-minute values to the activity of the ^3^H-biotin (45 Ci/mmol) per mg of protein in each sample. Apparent K(m) and V(max) values were obtained by plotting the data in the GraphPad Prism 5 (GraphPad Software, Inc) Michaelis-Menten enzyme kinetics template using non-linear regression analysis. Experiments to determine kinetic parameters were performed in triplicate with three replicates per individual experiment. Other experiments were performed in duplicate with three replicates per individual experiment.

Competitor studies were performed using samples prepared identically to uptake experiments. Samples were then incubated simultaneously with ^3^H-biotin and inhibitor (lipoic acid, L-methionine, biotin, or desthiobiotin [all from Sigma]) or no inhibitor for 1 minute at 30°C. Reactions were stopped and quantified as described for transport assays.

### SMVT immunofluorescence localization studies

HeLa or McCoy cells were grown until subconfluent on 35 mm glass bottom cell culture dishes (World Precision Instruments, Inc). Cells were then infected with *Chlamydia* at an MOI ∼0.1 and incubated for 20 to 24 hours prior to fixing in 3% paraformaldehyde/3% sucrose/PBS and permeablized with 0.2% Triton X-100/PBS. Prior to antibody treatment, samples were blocked in 10% FBS/DMEM. SMVT localization was assessed using either SMVT H56 (Santa Cruz, rabbit polyclonal against C-terminal peptide, used at 1∶100) or SMVT N14 (Santa Cruz, goat polyclonal against N-terminal peptide, used at 1∶100). For *C. trachomatis* 434/Bu infections, IncA was detected using a mouse anti-IncA antibody (from Ted Hackstadt, Rockey Mountain Laboratories, used at 1∶100). AlexaFlour 594 chicken anti-goat, AlexaFlour 594 goat anti-rabbit, and AlexaFlour 488 goat anti-mouse (all from Life Technologies) were used at a concentration of 1∶1000. Images were obtained using an Olympus BX51 microscope fitted with an Olympus DP-70 digital camera. Merged images were created using DP controller/manager software version 1.1.1.71.

### Bioinformatics

Analysis of biotin synthesis and fatty acid synthesis pathways was performed using KEGG PATHWAY maps (http://www.kegg.com/kegg/pathway.html). Genome synteny analysis was performed using the Genome Region Comparison tool from JCVI CMR (http://cmr.jcvi.org/cgi-bin/CMR/shared/MakeFrontPages.cgi?page=GenomeRegionComparison) and the NCBI genome database (http://www.ncbi.nlm.nih.gov/sites/genome). Protein homology searches were performed using NCBI blastp (default settings). E-values ≤1e^−10^ were considered significant for homology analysis. Proteins were aligned using ClustalW2 through the EMBL-EBI server (http://www.ebi.ac.uk/clustalw/) with default settings and the results were used to report percent amino acid sequence identity and guide trees using neighbor joining analysis. ClustalW2 alignment files were modified using BioEdit for visual sequence comparison [76].

### Sequencing of *bioF_1*

To confirm the genomic sequence data for the start sites of *bioF_1*, the 5′ regions of *bioF_1* from *C. pneumoniae* TWAR (CpB0956, NC_000922.1) and *C. trachomatis* 434/Bu (CTL0146, NC010287.1) were amplified using primers bioF1cpF (5′-GTTCCCTAGT GGTGTGTGGTATTCC-3′) and bioF1cpR (5′-CGATAACTGAAGAAGGCCCTACC-3′) or bioF1ctF (5′-AAATGGTTAGCCTTGAGGCCATGGAG-3′) and bioF1ctR (5′-ACCTGCTCGT CCCATAGCACATAATCC-3′), respectively. Genomic template was obtained using the QIAgen DNeasy kit and EBs from *C. pneumoniae* TW-183 or *C. trachomatis* 434/Bu. DNA was PCR amplified using Fermentas PCR Master Mix (Thermo Scientific) as directed. PCR products were purified using the MinElute PCR purification kit from QIAgen and directly sequenced in both directions with the same primers used for the PCR reaction. Samples were sequenced at the Uniformed Services University Biomedical Instrumentation Center.

## Supporting Information

Figure S1 **AccB sequence comparison.** AccB from *E. coli* and select *Chlamydia* (listed at the end of the alignment plot) were aligned using ClustalW. Identical (black shading) and similar (grey shading) amino acids are shown using a 100% sequence identity cut-off. The biotinylated lysine residue is shown in green. A neighbor joining tree with distances is shown in panel (B).
(TIFF)

Figure S2 **BirA sequences comparison.** BirA sequences from select *Chlamydia* (listed at the end of the alignment plot) were aligned with BirA from *E. coli* using ClustalW. Identical (black shading) and similar (grey shading) amino acids are shown using a 100% sequence identity cut-off. The helix-turn-helix DNA-binding region of the *E. coli* Class II BirA is highlighted in yellow. The BPL-ligase domain is shown in blue and the conserved biotin protein ligase C-terminal region is shown in green. A neighbor joining tree with distances is shown in panel (B).
(TIFF)

Figure S3 **BioF_1 and BioF_2 sequence comparisons.** BioF_1 and BioF_2 sequences were aligned using ClustalW. Identical (black shading) and similar (grey shading) amino acids are shown using a 100% sequence identity cut-off. The organisms used for analysis are shown at the end of the sequence plot. Residues marked in yellow indicate amino acids important for activity (for the *E. coli* BioF) that show variance across the chlamydial BioF_1 and BioF_2 sequences while those in green indicate conserved active site residues. Blue shading indicates active site amino acids that cluster separately between BioF_1 and BioF_2. Green shading indicates the conserved, catalytic lysine residue. Organisms shown in red font use a TTG initiation codon for *bioF_1*. A neighbor joining tree with distances is shown in panel (B).
(TIFF)

Figure S4 **BioY sequence comparison.** An alignment of BioY transporters was created using ClustalW. The bacteria species used are listed to the right of the plot (green font indicates organisms with functionally validated type I ECF biotin transporters). Percent amino acid identity versus the *Rhodobacter capsulatus* BioY is shown to the right of the second strand in the plot. Identical (black shading) and similar (grey shading) amino acids are shown using a 60% sequence identity cut-off. A neighbor joining tree with distances is shown in panel (B).
(TIFF)

Figure S5 **Expression of BioY in *E. coli*** **ATM1172.** Bacteria were grown with or without the inducer IPTG, lysed in Laemmli buffer and run on 12% SDS-PAGE gels for anti-His-tag Western blotting (A) or stained with Coomassie Brilliant blue (B). Different amounts of lysate were run in lanes 2–5 (10 µl) and lanes 7–10 (30 µl) to detect monomeric BioY. The position of the predicted BioY monomer and BioY dimer are indicated with black arrows to the right of panel (A). Lanes 1 and 6 in panel A and lane 1 in panel B contain molecular weight markers. The presence of IPTG during growth is indicated at the bottom of panels A and B. Panel A was made by merging the chemiluminescent image with a light image taken at the same magnification. Contrast and brightness of the merged image in panel A were uniformly adjusted to visualize the ∼20 kDa band in lane 10.
(TIFF)

Figure S6 **Transport of ^3^H-biotin by *E. coli*** **ATM1172 and *E. coli*** **ATM1173.** ^3^H-biotin transport was measured for *E. coli* ATM1172 (pET19b::*bioY*, His-tag) and *E. coli* ATM1173 (pET19b::*bioY*, no His-tag) versus *E. coli* ATM1135 (pET19b). In (A), bacteria were incubated with 200 nM ^3^H-biotin at 30°C for two minutes. In panel (B), bacteria were incubated with 100 nM ^3^H-biotin for 1, 2, 5, 10, or 30 minutes at 30°C. In panel (C), *E. coli* ATM1172 was incubated with 0.5, 1, 5, 10, 50, or 100 nM ^3^H-biotin at 30°C for 0.5, 1, or 3 minutes. Reactions were halted and radioactivity was measured using a scintillation counter.
(TIFF)

Figure S7 **Inhibition of ^3^H-biotin transport in *E. coli*** **ATM1172 and *E. coli*** **ATM1173.** The effect of various inhibitors on ^3^H-biotin uptake was tested by incubating samples with either 100 nM ^3^H-biotin and 10 µM biotin (A) or 50 nM ^3^H-biotin and 1 µM biotin, L-methionine, or lipoic acid (B and C). Panels B and C show results for experiments using *E. coli* ATM1172 and *E. coli* ATM1173, respectively. Samples were incubated at 30°C for 1 minute. Reactions were halted and radioactivity was measured using a scintillation counter. Values are reported as percent inhibition versus a no inhibitor control.
(TIFF)

Figure S8 **Localization of SMVT in infected HeLa cells using immunofluorescence microscopy.** HeLa cells were mock infected (A) or infected (B and C) for 20–24 hours, fixed and permeablized, and probed with anti-IncA antibodies (panels A and B, shown in green) and anti-SMVT N14 antibodies (A and B) or only anti-SMVT H56 antibodies (C) (shown in red). The location of inclusions viewed under phase contrast (top panel, A and B) and fluorescence (middle and bottom panels B) are shown with white arrows. The inset in panel B was created by merging the red and green channel micrographs. Cells were infected with *C. trachomatis* 434/Bu (panel B), or *C trachomatis* serovar E (CtE), *C. caviae* (Cc), or *C. psitaci* (Cps) in panel C. Phase contrast and red channel (SMVT H56) micrographs were merged for panel C images and an example inclusion is marked with white arrows in each panel.
(TIFF)

Figure S9 **Localization of SMVT in infected McCoy cells using immunofluorescence microscopy.** McCoy cells were infected for 20–24 hours, fixed and permeablized, and probed with anti-SMVT (H56) antibodies (shown in red) and anti-IncA antibodies (shown in green). The inset in panel C was created by merging the red and green channel micrographs. The SMVT H56 antibody was raised against a C-terminal peptide from the human SMVT (amino acids 528–580). Species cross reactivity between the human and mouse SMVT was expected with H56 because the mouse peptide region shares 49/52 amino acids with the human antigenic peptide region.
(TIFF)

Table S1 **Biotin acquisition and utilization genes in obligate intracellular bacteria.**
(XLSX)

Table S2 **K(m) and V(max) values for bacterial biotin transporters.**
(XLSX)

Table S3 **List of cell lines and bacterial strains used in this study.**
(XLSX)

## References

[1] Johnson DL, Mahony JB (2007) C*hlamydophila pneumoniae* PknD exhibits dual amino acid specificity and phosphorylates Cpn0712, a putative type III secretion YscD homolog. J Bacteriol 189: 7549–7555. 17766419 10.1128/JB.00893-07 PMC2168749

[2] Kalman S, Mitchell W, Marathe R, Lammel C, Fan J, et al (1999) Comparative genomes of *Chlamydia pneumoniae* and *C. trachomatis* . Nat Genet 21: 385–389. 10192388 10.1038/7716

[3] Baneke A (2012) Review: Targeting trachoma: Strategies to reduce the leading infectious cause of blindness. Travel Med Infect Dis 10.1016/j.tmaid.2012.01.005 22326056

[4] Miller KE (2006) Diagnosis and treatment of *Chlamydia trachomatis* infection. Am Fam Physician 73: 1411–1416. 16669564

[5] Longbottom D, Livingstone M (2006) Vaccination against chlamydial infections of man and animals. Vet J 171: 263–275. 16490708 10.1016/j.tvjl.2004.09.006

[6] Abdelrahman YM, Belland RJ (2005) The chlamydial developmental cycle. FEMS Microbiol Rev 29: 949–959. 16043254 10.1016/j.femsre.2005.03.002

[7] Peters J, Wilson DP, Myers G, Timms P, Bavoil PM (2007) Type III secretion a la *Chlamydia* . Trends Microbiol 15: 241–251. 17482820 10.1016/j.tim.2007.04.005

[8] Saka HA, Valdivia RH (2010) Acquisition of nutrients by *Chlamydiae*: unique challenges of living in an intracellular compartment. Curr Opin Microbiol 13: 4–10. 20006538 10.1016/j.mib.2009.11.002 PMC3202608

[9] Byrne GI (2010) *Chlamydia trachomatis* strains and virulence: rethinking links to infection prevalence and disease severity. J Infect Dis 201 Suppl 2: S126–133. 20470049 10.1086/652398 PMC2878587

[10] Horn M, Collingro A, Schmitz-Esser S, Beier CL, Purkhold U, et al (2004) Illuminating the evolutionary history of chlamydiae. Science 304: 728–730. 15073324 10.1126/science.1096330

[11] Voigt A, Schofl G, Saluz HP (2012) The *Chlamydia psittaci* Genome: A Comparative Analysis of Intracellular Pathogens. PLoS One 7: e35097. 22506068 10.1371/journal.pone.0035097 PMC3323650

[12] Stephens RS, Kalman S, Lammel C, Fan J, Marathe R, et al (1998) Genome sequence of an obligate intracellular pathogen of humans: *Chlamydia trachomatis* . Science 282: 754–759. 9784136 10.1126/science.282.5389.754

[13] McClarty G, Caldwell HD, Nelson DE (2007) Chlamydial interferon gamma immune evasion influences infection tropism. Curr Opin Microbiol 10: 47–51. 17208039 10.1016/j.mib.2006.12.003

[14] Aziz RK, Kansal R, Aronow BJ, Taylor WL, Rowe SL, et al (2010) Microevolution of group A streptococci in vivo: capturing regulatory networks engaged in sociomicrobiology, niche adaptation, and hypervirulence. PLoS One 5: e9798. 20418946 10.1371/journal.pone.0009798 PMC2854683

[15] Barelle CJ, Priest CL, Maccallum DM, Gow NA, Odds FC, et al (2006) Niche-specific regulation of central metabolic pathways in a fungal pathogen. Cell Microbiol 8: 961–971. 16681837 10.1111/j.1462-5822.2005.00676.x PMC1472618

[16] Deng X, Phillippy AM, Li Z, Salzberg SL, Zhang W (2010) Probing the pan-genome of *Listeria monocytogenes*: new insights into intraspecific niche expansion and genomic diversification. BMC Genomics 11: 500. 20846431 10.1186/1471-2164-11-500 PMC2996996

[17] Letek M, Gonzalez P, Macarthur I, Rodriguez H, Freeman TC, et al (2010) The genome of a pathogenic rhodococcus: cooptive virulence underpinned by key gene acquisitions. PLoS Genet 6. 10.1371/journal.pgen.1001145 PMC2947987 20941392

[18] Ward PN, Holden MT, Leigh JA, Lennard N, Bignell A, et al (2009) Evidence for niche adaptation in the genome of the bovine pathogen *Streptococcus uberis* . BMC Genomics 10: 54. 19175920 10.1186/1471-2164-10-54 PMC2657157

[19] Zempleni J, Wijeratne SS, Hassan YI (2009) Biotin. Biofactors 35: 36–46. 19319844 10.1002/biof.8 PMC4757853

[20] Purushothaman S, Annamalai K, Tyagi AK, Surolia A (2011) Diversity in functional organization of class I and class II biotin protein ligase. PLoS One 6: e16850. 21390227 10.1371/journal.pone.0016850 PMC3048393

[21] Zempleni J (2005) Uptake, localization, and noncarboxylase roles of biotin. Annu Rev Nutr 25: 175–196. 16011464 10.1146/annurev.nutr.25.121304.131724

[22] Lin S, Cronan JE (2011) Closing in on complete pathways of biotin biosynthesis. Mol Biosyst 7: 1811–1821. 21437340 10.1039/c1mb05022b

[23] Streit WR, Entcheva P (2003) Biotin in microbes, the genes involved in its biosynthesis, its biochemical role and perspectives for biotechnological production. Appl Microbiol Biotechnol 61: 21–31. 12658511 10.1007/s00253-002-1186-2

[24] Hebbeln P, Rodionov DA, Alfandega A, Eitinger T (2007) Biotin uptake in prokaryotes by solute transporters with an optional ATP-binding cassette-containing module. Proc Natl Acad Sci U S A 104: 2909–2914. 17301237 10.1073/pnas.0609905104 PMC1815280

[25] Prakash O, Eisenberg MA (1974) Active transport of biotin in *Escherichia coli* K-12. J Bacteriol 120: 785–791. 4616949 10.1128/jb.120.2.785-791.1974 PMC245839

[26] Rodionov DA, Hebbeln P, Eudes A, ter Beek J, Rodionova IA, et al (2009) A novel class of modular transporters for vitamins in prokaryotes. J Bacteriol 191: 42–51. 18931129 10.1128/JB.01208-08 PMC2612444

[27] Prasad PD, Wang H, Kekuda R, Fujita T, Fei YJ, et al (1998) Cloning and functional expression of a cDNA encoding a mammalian sodium-dependent vitamin transporter mediating the uptake of pantothenate, biotin, and lipoate. J Biol Chem 273: 7501–7506. 9516450 10.1074/jbc.273.13.7501

[28] Ramaswamy AV, Maurelli AT (2010) *Chlamydia trachomatis* serovar L2 can utilize exogenous lipoic acid through the action of the lipoic acid ligase LplA1. J Bacteriol 192: 6172–6181. 20870766 10.1128/JB.00717-10 PMC2981205

[29] Polyak SW, Abell AD, Wilce MC, Zhang L, Booker GW (2012) Structure, function and selective inhibition of bacterial acetyl-coa carboxylase. Appl Microbiol Biotechnol 93: 983–992. 22183085 10.1007/s00253-011-3796-z

[30] Choi-Rhee E, Schulman H, Cronan JE (2004) Promiscuous protein biotinylation by *Escherichia coli* biotin protein ligase. Protein Sci 13: 3043–3050. 15459338 10.1110/ps.04911804 PMC2286582

[31] Zempleni J, Hassan YI, Wijeratne SS (2008) Biotin and biotinidase deficiency. Expert Rev Endocrinol Metab 3: 715–724. 19727438 10.1586/17446651.3.6.715 PMC2726758

[32] Rodionov DA, Mironov AA, Gelfand MS (2002) Conservation of the biotin regulon and the BirA regulatory signal in Eubacteria and Archaea. Genome Res 12: 1507–1516. 12368242 10.1101/gr.314502 PMC187538

[33] Dugan J, Rockey DD, Jones L, Andersen AA (2004) Tetracycline resistance in *Chlamydia suis* mediated by genomic islands inserted into the chlamydial inv-like gene. Antimicrob Agents Chemother 48: 3989–3995. 15388463 10.1128/AAC.48.10.3989-3995.2004 PMC521927

[34] Sait M, Clark EM, Wheelhouse N, Livingstone M, Spalding L, et al (2011) Genome sequence of the *Chlamydophila abortus* variant strain LLG. J Bacteriol 193: 4276–4277. 21685275 10.1128/JB.05290-11 PMC3147691

[35] Webster SP, Alexeev D, Campopiano DJ, Watt RM, Alexeeva M, et al (2000) Mechanism of 8-amino-7-oxononanoate synthase: spectroscopic, kinetic, and crystallographic studies. Biochemistry 39: 516–528. 10642176 10.1021/bi991620j

[36] Guillen-Navarro K, Araiza G, Garcia-de los Santos A, Mora Y, Dunn MF (2005) The *Rhizobium etli* bioMNY operon is involved in biotin transport. FEMS Microbiol Lett 250: 209–219. 16099603 10.1016/j.femsle.2005.07.020

[37] Schneider J, Peters-Wendisch P, Stansen KC, Gotker S, Maximow S, et al (2012) Characterization of the biotin uptake system encoded by the biotin-inducible bioYMN operon of *Corynebacterium glutamicum* . BMC Microbiol 12: 6. 22243621 10.1186/1471-2180-12-6 PMC3398298

[38] Entcheva P, Phillips DA, Streit WR (2002) Functional analysis of *Sinorhizobium melilot*i genes involved in biotin synthesis and transport. Appl Environ Microbiol 68: 2843–2848. 12039741 10.1128/AEM.68.6.2843-2848.2002 PMC123963

[39] Miroux B, Walker JE (1996) Over-production of proteins in *Escherichia coli*: mutant hosts that allow synthesis of some membrane proteins and globular proteins at high levels. J Mol Biol 260: 289–298. 8757792 10.1006/jmbi.1996.0399

[40] Finkenwirth F, Neubauer O, Gunzenhauser J, Schoknecht J, Scolari S, et al (2010) Subunit composition of an energy-coupling-factor-type biotin transporter analysed in living bacteria. Biochem J 431: 373–380. 20738254 10.1042/BJ20100813

[41] Grieshaber S, Swanson JA, Hackstadt T (2002) Determination of the physical environment within the *Chlamydia trachomatis* inclusion using ion-selective ratiometric probes. Cell Microbiol 4: 273–283. 12027956 10.1046/j.1462-5822.2002.00191.x

[42] Heinzen RA, Hackstadt T (1997) The *Chlamydia trachomatis* parasitophorous vacuolar membrane is not passively permeable to low-molecular-weight compounds. Infect Immun 65: 1088–1094. 9038320 10.1128/iai.65.3.1088-1094.1997 PMC175092

[43] Horn M (2008) *Chlamydiae* as symbionts in eukaryotes. Annu Rev Microbiol 62: 113–131. 18473699 10.1146/annurev.micro.62.081307.162818

[44] Giles TN, Fisher DJ, Graham DE (2009) Independent inactivation of arginine decarboxylase genes by nonsense and missense mutations led to pseudogene formation in *Chlamydia trachomatis* serovar L2 and D strains. BMC Evol Biol 9: 166. 19607664 10.1186/1471-2148-9-166 PMC2720952

[45] Said HM (2012) Biotin: biochemical, physiological and clinical aspects. Subcell Biochem 56: 1–19. 22116691 10.1007/978-94-007-2199-9_1

[46] Said HM, Ortiz A, McCloud E, Dyer D, Moyer MP, et al (1998) Biotin uptake by human colonic epithelial NCM460 cells: a carrier-mediated process shared with pantothenic acid. Am J Physiol 275: C1365–1371. 9814986 10.1152/ajpcell.1998.275.5.C1365

[47] Delli-Bovi TA, Spalding MD, Prigge ST (2010) Overexpression of biotin synthase and biotin ligase is required for efficient generation of sulfur-35 labeled biotin in *E. coli* . BMC Biotechnol 10: 73. 20937134 10.1186/1472-6750-10-73 PMC2964542

[48] Soriano A, Radice AD, Herbitter AH, Langsdorf EF, Stafford JM, et al (2006) *Escherichia coli* acetyl-coenzyme A carboxylase: characterization and development of a high-throughput assay. Anal Biochem 349: 268–276. 16325142 10.1016/j.ab.2005.10.044

[49] Shaw AC, Gevaert K, Demol H, Hoorelbeke B, Vandekerckhove J, et al (2002) Comparative proteome analysis of *Chlamydia trachomatis* serovar A, D and L2. Proteomics 2: 164–186. 11840563 10.1002/1615-9861(200202)2:2<164::aid-prot164>3.0.co;2-u

[50] Kuroishi T, Rios-Avila L, Pestinger V, Wijeratne SS, Zempleni J (2011) Biotinylation is a natural, albeit rare, modification of human histones. Mol Genet Metab 104: 537–545. 21930408 10.1016/j.ymgme.2011.08.030 PMC3224183

[51] Saka HA, Thompson JW, Chen YS, Kumar Y, Dubois LG, et al (2011) Quantitative proteomics reveals metabolic and pathogenic properties of *Chlamydia trachomatis* developmental forms. Mol Microbiol 82: 1185–1203. 22014092 10.1111/j.1365-2958.2011.07877.x PMC3225693

[52] Vandahl BB, Birkelund S, Christiansen G (2002) Proteome analysis of *Chlamydia pneumoniae* . Methods Enzymol 358: 277–288. 12474393 10.1016/s0076-6879(02)58095-2

[53] Wylie JL, Hatch GM, McClarty G (1997) Host cell phospholipids are trafficked to and then modified by *Chlamydia trachomatis* . J Bacteriol 179: 7233–7242. 9393685 10.1128/jb.179.23.7233-7242.1997 PMC179671

[54] Belland RJ, Zhong G, Crane DD, Hogan D, Sturdevant D, et al (2003) Genomic transcriptional profiling of the developmental cycle of *Chlamydia trachomatis* . Proc Natl Acad Sci U S A 100: 8478–8483. 12815105 10.1073/pnas.1331135100 PMC166254

[55] Maurer AP, Mehlitz A, Mollenkopf HJ, Meyer TF (2007) Gene Expression Profiles of *Chlamydophila pneumoniae* during the Developmental Cycle and Iron Depletion-Mediated Persistence. PLoS Pathog 3: e83. 17590080 10.1371/journal.ppat.0030083 PMC1894823

[56] Dakshinamurti K, Mistry SP (1963) Tissue and intracellular distribution of biotin-C-1400H in rats and chicks. J Biol Chem 238: 294–296. 14024685

[57] Mock DM (2005) Marginal biotin deficiency is teratogenic in mice and perhaps humans: a review of biotin deficiency during human pregnancy and effects of biotin deficiency on gene expression and enzyme activities in mouse dam and fetus. J Nutr Biochem 16: 435–437. 15992686 10.1016/j.jnutbio.2005.03.022

[58] Taniguchi A, Watanabe T (2008) Transplacental transport and tissue distribution of biotin in mice at midgestation. Congenit Anom (Kyoto) 48: 57–62. 18452485 10.1111/j.1741-4520.2008.00179.x

[59] Bouakane A, Benchaieb I, Rodolakis A (2003) Abortive potency of *Chlamydophila abortus* in pregnant mice is not directly correlated with placental and fetal colonization levels. Infect Immun 71: 7219–7222. 14638821 10.1128/IAI.71.12.7219-7222.2003 PMC308942

[60] Sullivan JT, Brown SD, Yocum RR, Ronson CW (2001) The bio operon on the acquired symbiosis island of *Mesorhizobium* sp. strain R7A includes a novel gene involved in pimeloyl-CoA synthesis. Microbiology 147: 1315–1322. 11320134 10.1099/00221287-147-5-1315

[61] Belunis CJ, Mdluli KE, Raetz CR, Nano FE (1992) A novel 3-deoxy-D-manno-octulosonic acid transferase from *Chlamydia trachomatis* required for expression of the genus-specific epitope. J Biol Chem 267: 18702–18707. 1382060

[62] McCoy AJ, Maurelli AT (2005) Characterization of *Chlamydia* MurC-Ddl, a fusion protein exhibiting D-alanyl-D-alanine ligase activity involved in peptidoglycan synthesis and D-cycloserine sensitivity. Mol Microbiol 57: 41–52. 15948948 10.1111/j.1365-2958.2005.04661.x

[63] Waller JR, Lichstein HC (1965) Biotin transport and accumulation by cells of *Lactobacillus plantarum*. II. Kinetics of the system. J Bacteriol 90: 853–856. 5847806 10.1128/jb.90.4.853-856.1965 PMC315748

[64] Neubauer O, Reiffler C, Behrendt L, Eitinger T (2011) Interactions among the A and T units of an ECF-type biotin transporter analyzed by site-specific crosslinking. PLoS One 6: e29087. 22216173 10.1371/journal.pone.0029087 PMC3246461

[65] Berntsson RP, Ter Beek J, Majsnerowska M, Duurkens RH, Puri P, et al (2012) Structural divergence of paralogous S components from ECF-type ABC transporters. Proc Natl Acad Sci U S A 10.1073/pnas.1203219109 PMC3435211 22891302

[66] ter Beek J, Duurkens RH, Erkens GB, Slotboom DJ (2011) Quaternary structure and functional unit of energy coupling factor (ECF)-type transporters. J Biol Chem 286: 5471–5475. 21135102 10.1074/jbc.M110.199224 PMC3037660

[67] Cocchiaro JL, Valdivia RH (2009) New insights into *Chlamydia* intracellular survival mechanisms. Cell Microbiol 11: 1571–1578. 19673891 10.1111/j.1462-5822.2009.01364.x PMC2787098

[68] Elwell CA, Engel JN (2012) Lipid acquisition by intracellular *Chlamydiae* . Cell Microbiol 10.1111/j.1462-5822.2012.01794.x PMC3376245 22452394

[69] Valdivia RH (2008) Chlamydia effector proteins and new insights into chlamydial cellular microbiology. Curr Opin Microbiol 11: 53–59. 18299248 10.1016/j.mib.2008.01.003

[70] Grieshaber SS, Grieshaber NA, Hackstadt T (2003) *Chlamydia trachomatis* uses host cell dynein to traffic to the microtubule-organizing center in a p50 dynamitin-independent process. J Cell Sci 116: 3793–3802. 12902405 10.1242/jcs.00695

[71] Cocchiaro JL, Kumar Y, Fischer ER, Hackstadt T, Valdivia RH (2008) Cytoplasmic lipid droplets are translocated into the lumen of the *Chlamydia trachomatis* parasitophorous vacuole. Proc Natl Acad Sci U S A 105: 9379–9384. 18591669 10.1073/pnas.0712241105 PMC2453745

[72] Hackstadt T, Rockey DD, Heinzen RA, Scidmore MA (1996) *Chlamydia trachomatis* interrupts an exocytic pathway to acquire endogenously synthesized sphingomyelin in transit from the Golgi apparatus to the plasma membrane. Embo J 15: 964–977. 8605892 PMC449991

[73] Elwell CA, Jiang S, Kim JH, Lee A, Wittmann T, et al (2011) *Chlamydia trachomatis* co-opts GBF1 and CERT to acquire host sphingomyelin for distinct roles during intracellular development. PLoS Pathog 7: e1002198. 21909260 10.1371/journal.ppat.1002198 PMC3164637

[74] Verbeke P, Welter-Stahl L, Ying S, Hansen J, Hacker G, et al (2006) Recruitment of BAD by the *Chlamydia trachomatis* vacuole correlates with host-cell survival. PLoS Pathog 2: e45. 16710454 10.1371/journal.ppat.0020045 PMC1463014

[75] Mital J, Miller NJ, Fischer ER, Hackstadt T (2010) Specific chlamydial inclusion membrane proteins associate with active Src family kinases in microdomains that interact with the host microtubule network. Cell Microbiol 12: 1235–1249. 20331642 10.1111/j.1462-5822.2010.01465.x PMC2923664

[76] Hall T (1999) BioEdit: a user-friendly biological sequence alignment editor and analysis program for Windows 95/98/NT. Nucl Acids Symp Ser 41: 95–98.

