# Supplementary figures and images for: Uptake of Biotin by Chlamydia Spp. through the Use of a Bacterial Transporter (BioY) and a Host-Cell Transporter (SMVT)
Source: PLoS One. 2012 Sep 27;7(9):e46052. doi: 10.1371/journal.pone.0046052 (PMC3459881; doi:10.1371/journal.pone.0046052)

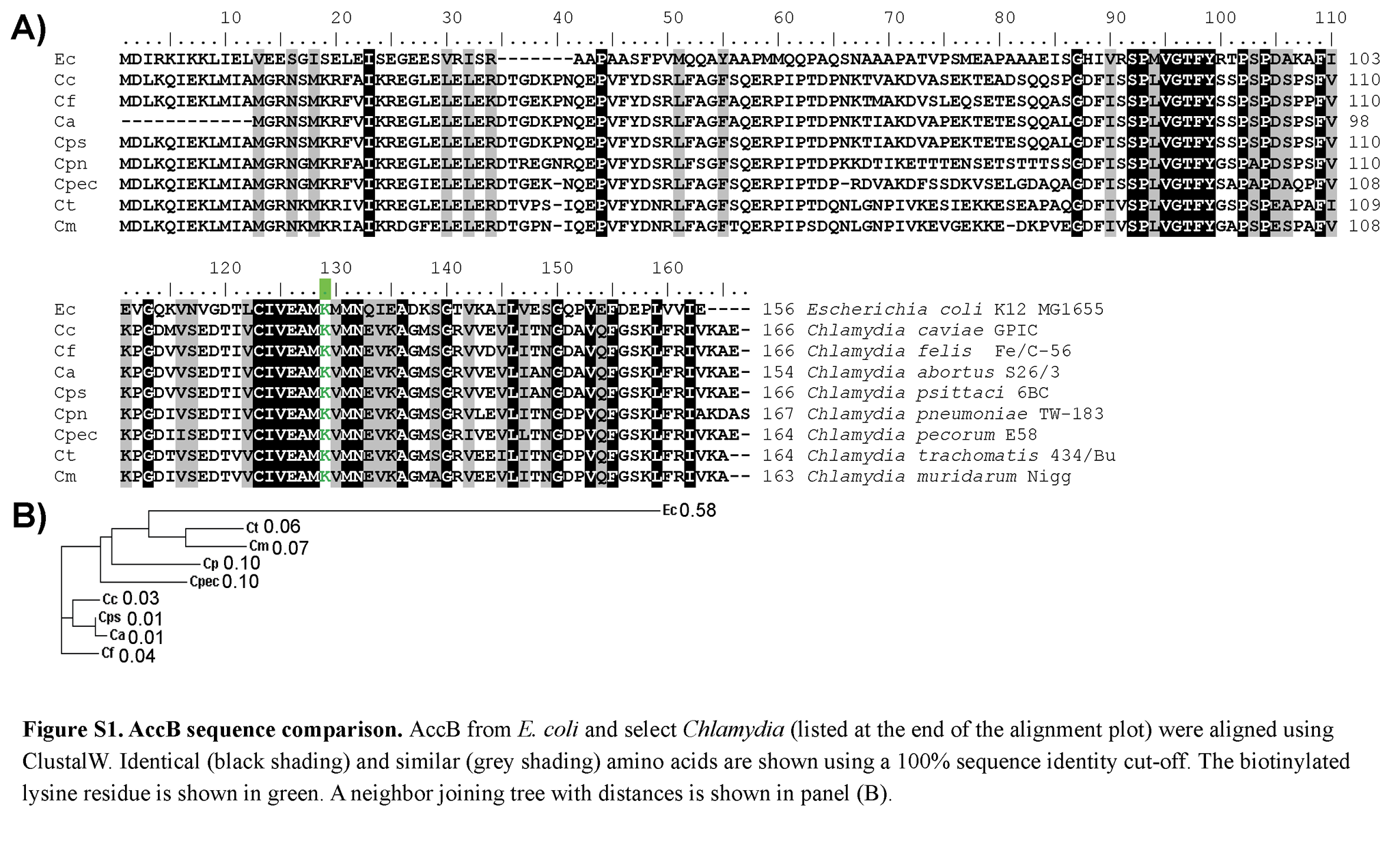

Supplement: Figure S1 — AccB sequence comparison. AccB from E. coli and select Chlamydia (listed at the end of the alignment plot) were aligned using ClustalW. Identical (black shading) and similar (grey shading) amino acids are shown using a 100% sequence identity cut-off. The biotinylated lysine residue is shown in green. A neighbor joining tree with distances is shown in panel (B). (TIFF) [file pone.0046052.s001.tiff]

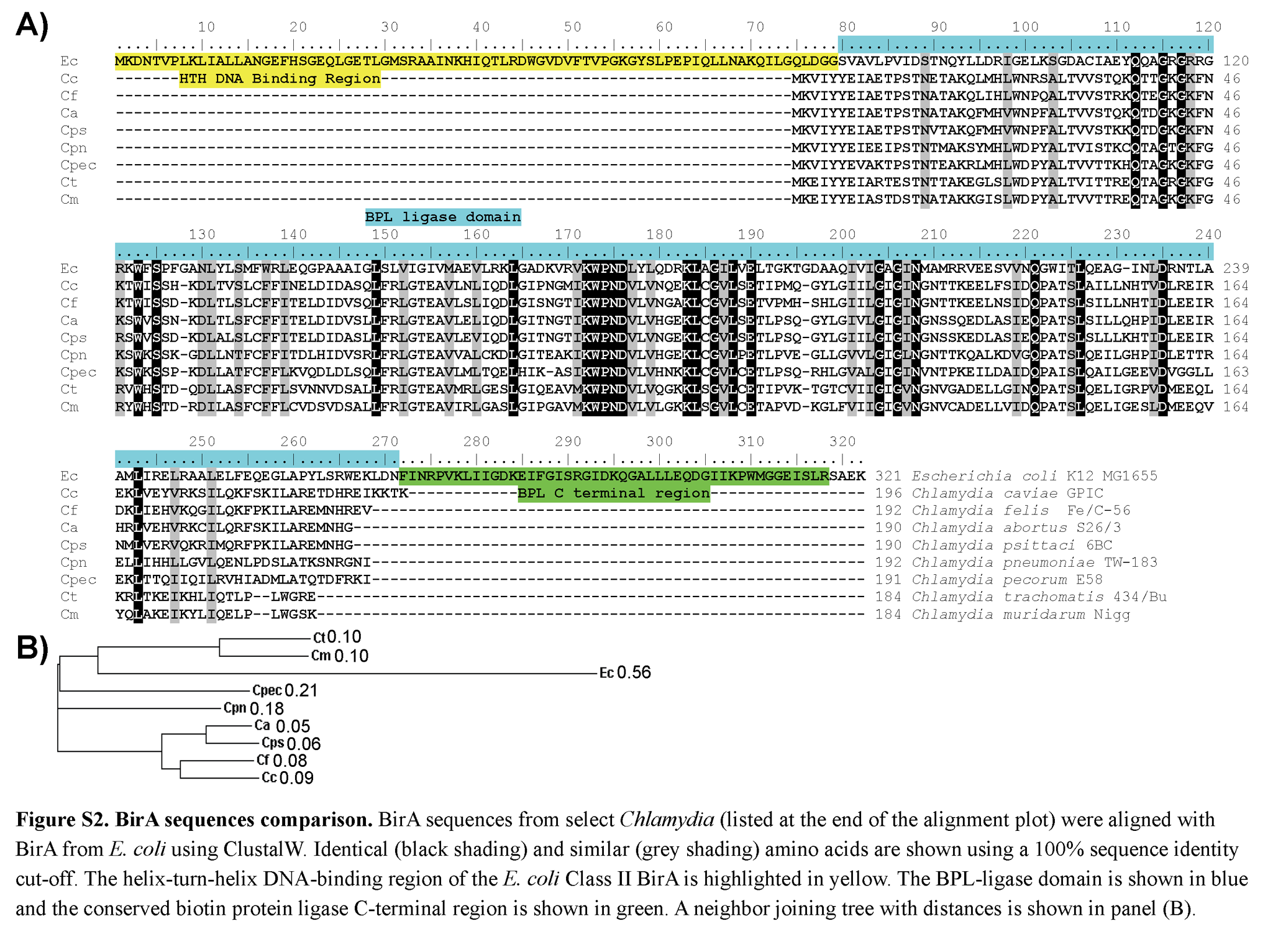

Supplement: Figure S2 — BirA sequences comparison. BirA sequences from select Chlamydia (listed at the end of the alignment plot) were aligned with BirA from E. coli using ClustalW. Identical (black shading) and similar (grey shading) amino acids are shown using a 100% sequence identity cut-off. The helix-turn-helix DNA-binding region of the E. coli Class II BirA is highlighted in yellow. The BPL-ligase domain is shown in blue and the conserved biotin protein ligase C-terminal region is shown in green. A neighbor joining tree with distances is shown in panel (B). (TIFF) [file pone.0046052.s002.tiff]

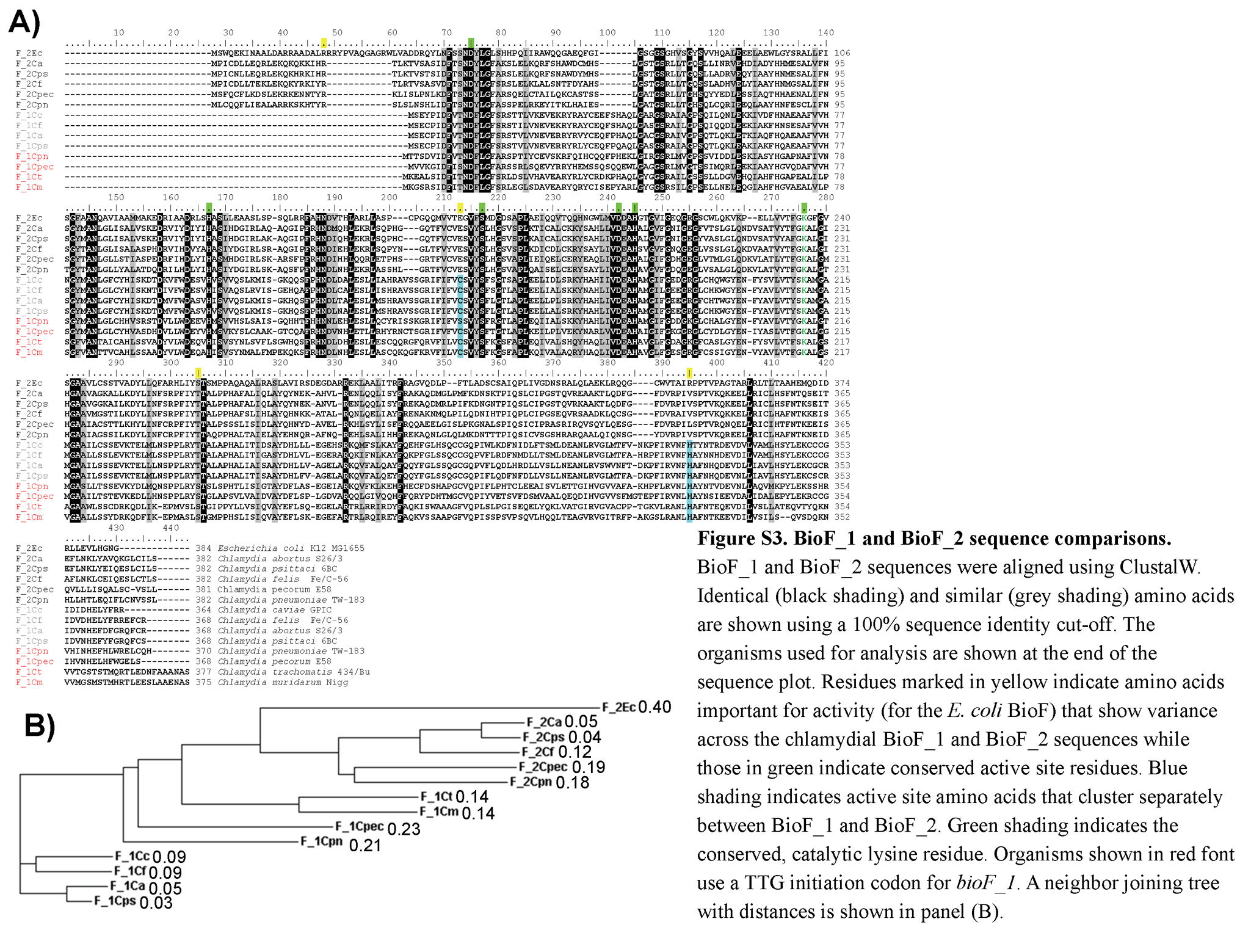

Supplement: Figure S3 — BioF_1 and BioF_2 sequence comparisons. BioF_1 and BioF_2 sequences were aligned using ClustalW. Identical (black shading) and similar (grey shading) amino acids are shown using a 100% sequence identity cut-off. The organisms used for analysis are shown at the end of the sequence plot. Residues marked in yellow indicate amino acids important for activity (for the E. coli BioF) that show variance across the chlamydial BioF_1 and BioF_2 sequences while those in green indicate conserved active site residues. Blue shading indicates active site amino acids that cluster separately between BioF_1 and BioF_2. Green shading indicates the conserved, catalytic lysine residue. Organisms shown in red font use a TTG initiation codon for bioF_1. A neighbor joining tree with distances is shown in panel (B). (TIFF) [file pone.0046052.s003.tiff]

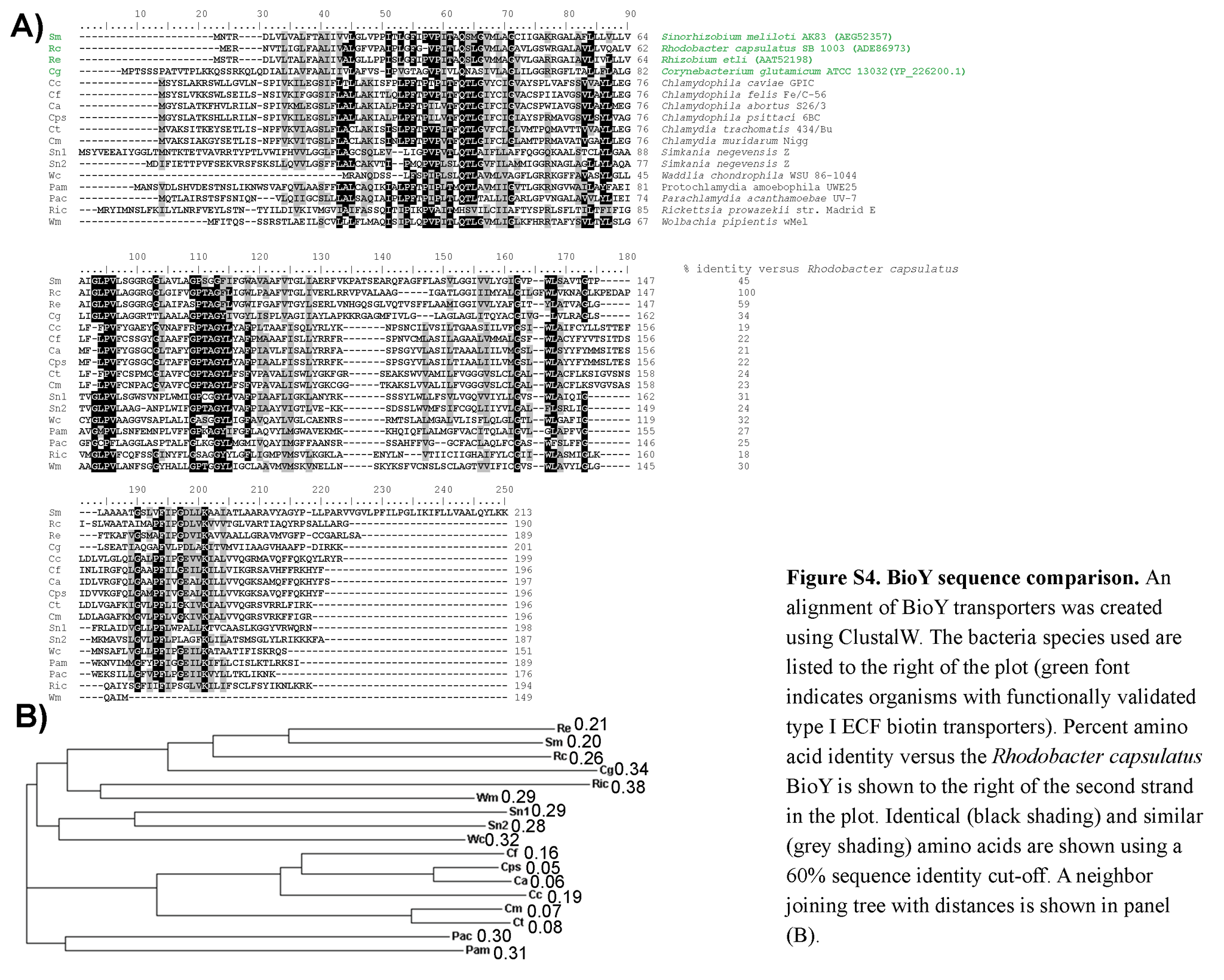

Supplement: Figure S4 — BioY sequence comparison. An alignment of BioY transporters was created using ClustalW. The bacteria species used are listed to the right of the plot (green font indicates organisms with functionally validated type I ECF biotin transporters). Percent amino acid identity versus the Rhodobacter capsulatus BioY is shown to the right of the second strand in the plot. Identical (black shading) and similar (grey shading) amino acids are shown using a 60% sequence identity cut-off. A neighbor joining tree with distances is shown in panel (B). (TIFF) [file pone.0046052.s004.tiff]

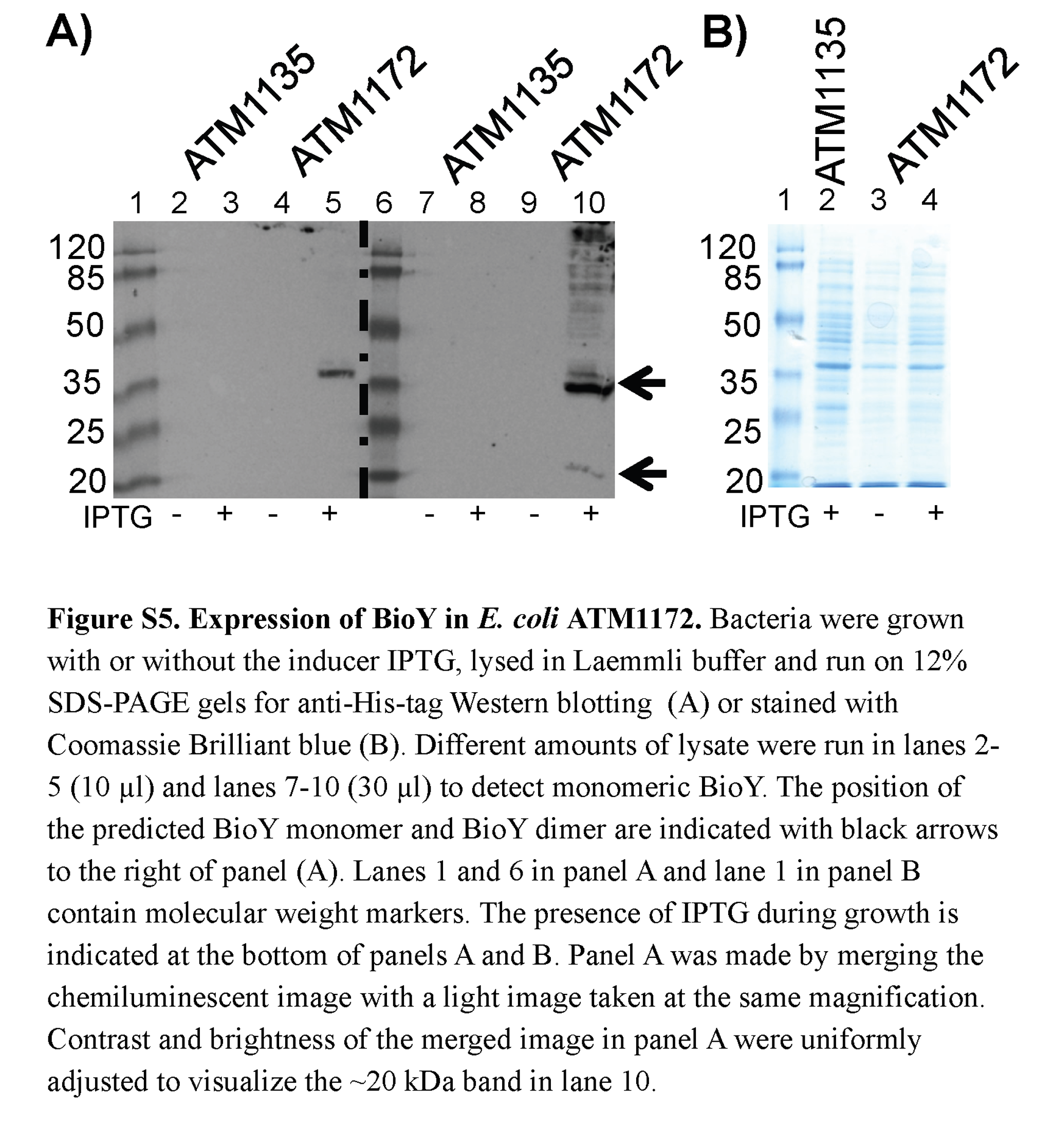

Supplement: Figure S5 — Expression of BioY in E. coli ATM1172. Bacteria were grown with or without the inducer IPTG, lysed in Laemmli buffer and run on 12% SDS-PAGE gels for anti-His-tag Western blotting (A) or stained with Coomassie Brilliant blue (B). Different amounts of lysate were run in lanes 2–5 (10 µl) and lanes 7–10 (30 µl) to detect monomeric BioY. The position of the predicted BioY monomer and BioY dimer are indicated with black arrows to the right of panel (A). Lanes 1 and 6 in panel A and lane 1 in panel B contain molecular weight markers. The presence of IPTG during growth is indicated at the bottom of panels A and B. Panel A was made by merging the chemiluminescent image with a light image taken at the same magnification. Contrast and brightness of the merged image in panel A were uniformly adjusted to visualize the ∼20 kDa band in lane 10. (TIFF) [file pone.0046052.s005.tiff]

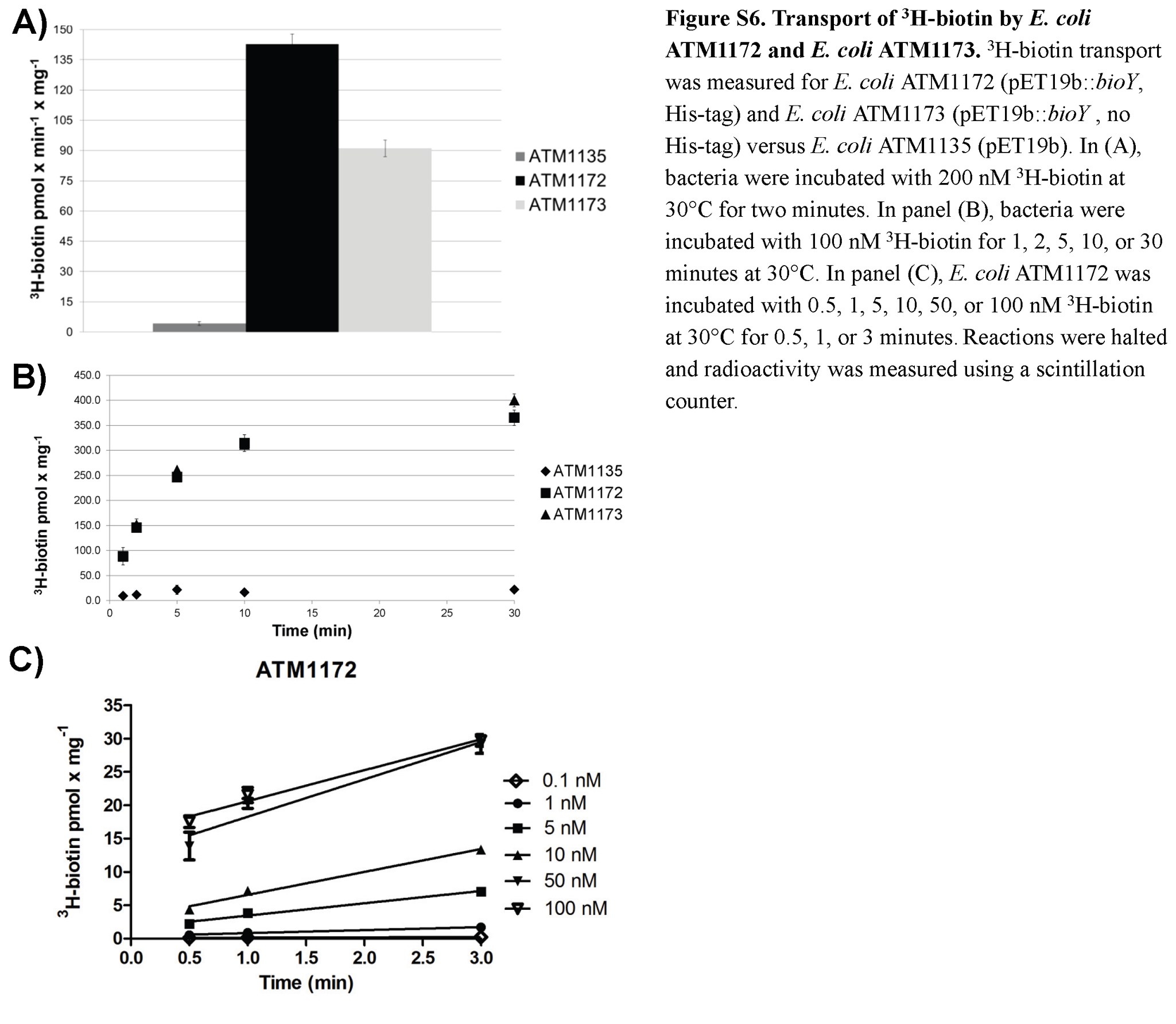

Supplement: Figure S6 — Transport of 3H-biotin by E. coli ATM1172 and E. coli ATM1173. 3H-biotin transport was measured for E. coli ATM1172 (pET19b::bioY, His-tag) and E. coli ATM1173 (pET19b::bioY, no His-tag) versus E. coli ATM1135 (pET19b). In (A), bacteria were incubated with 200 nM 3H-biotin at 30°C for two minutes. In panel (B), bacteria were incubated with 100 nM 3H-biotin for 1, 2, 5, 10, or 30 minutes at 30°C. In panel (C), E. coli ATM1172 was incubated with 0.5, 1, 5, 10, 50, or 100 nM 3H-biotin at 30°C for 0.5, 1, or 3 minutes. Reactions were halted and radioactivity was measured using a scintillation counter. (TIFF) [file pone.0046052.s006.tiff]

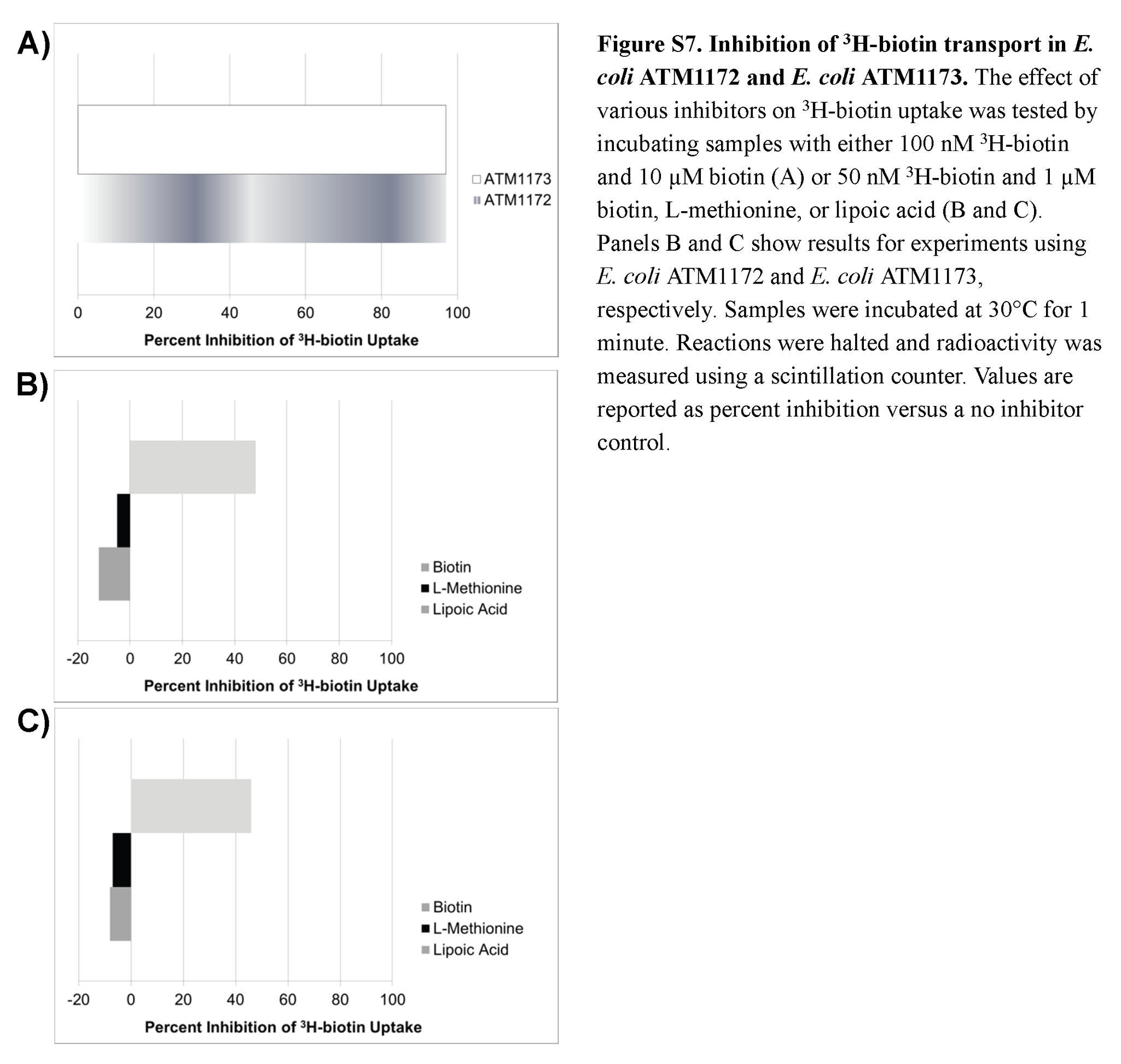

Supplement: Figure S7 — Inhibition of 3H-biotin transport in E. coli ATM1172 and E. coli ATM1173. The effect of various inhibitors on 3H-biotin uptake was tested by incubating samples with either 100 nM 3H-biotin and 10 µM biotin (A) or 50 nM 3H-biotin and 1 µM biotin, L-methionine, or lipoic acid (B and C). Panels B and C show results for experiments using E. coli ATM1172 and E. coli ATM1173, respectively. Samples were incubated at 30°C for 1 minute. Reactions were halted and radioactivity was measured using a scintillation counter. Values are reported as percent inhibition versus a no inhibitor control. (TIFF) [file pone.0046052.s007.tiff]

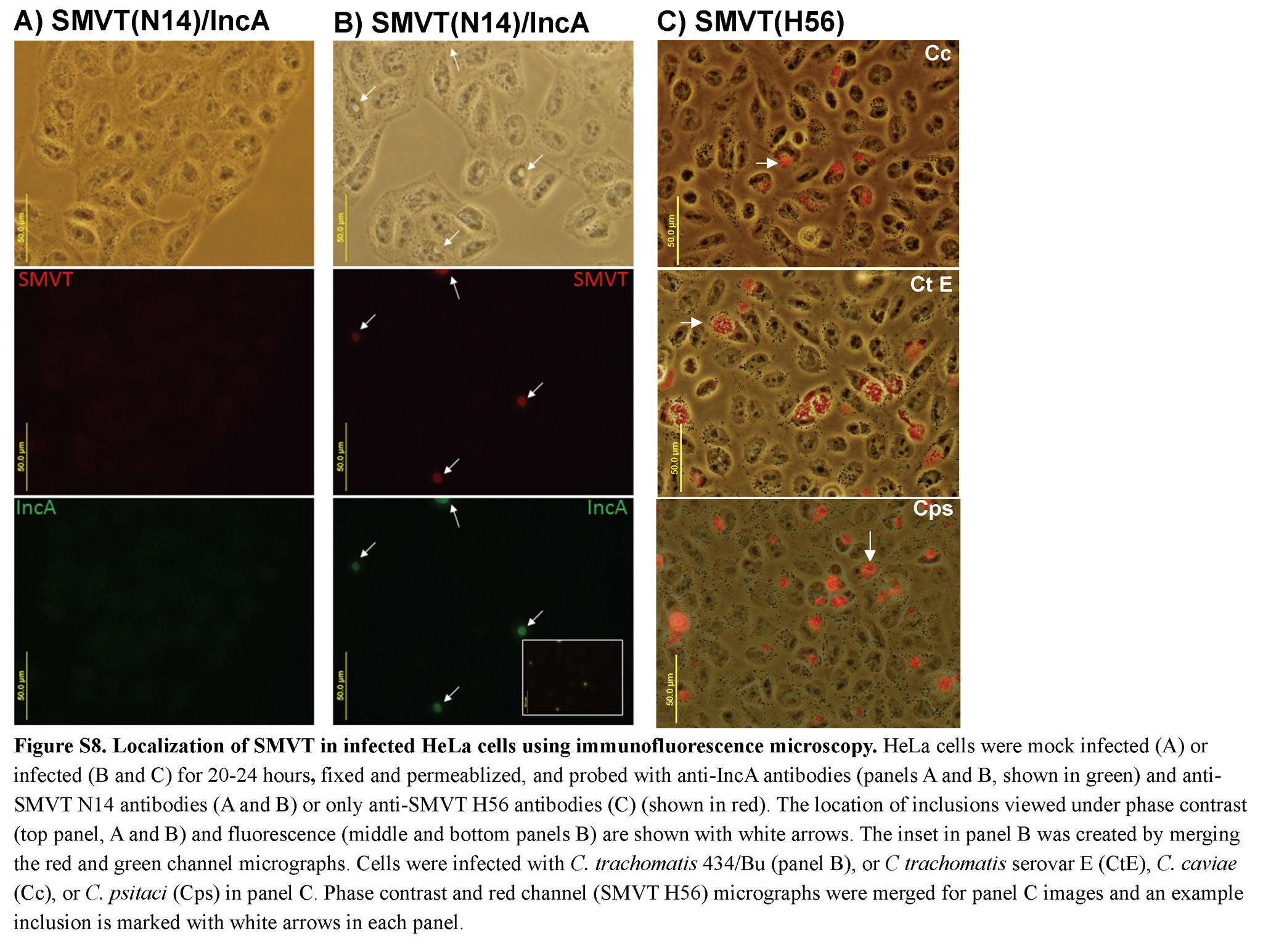

Supplement: Figure S8 — Localization of SMVT in infected HeLa cells using immunofluorescence microscopy. HeLa cells were mock infected (A) or infected (B and C) for 20–24 hours, fixed and permeablized, and probed with anti-IncA antibodies (panels A and B, shown in green) and anti-SMVT N14 antibodies (A and B) or only anti-SMVT H56 antibodies (C) (shown in red). The location of inclusions viewed under phase contrast (top panel, A and B) and fluorescence (middle and bottom panels B) are shown with white arrows. The inset in panel B was created by merging the red and green channel micrographs. Cells were infected with C. trachomatis 434/Bu (panel B), or C trachomatis serovar E (CtE), C. caviae (Cc), or C. psitaci (Cps) in panel C. Phase contrast and red channel (SMVT H56) micrographs were merged for panel C images and an example inclusion is marked with white arrows in each panel. (TIFF) [file pone.0046052.s008.tiff]

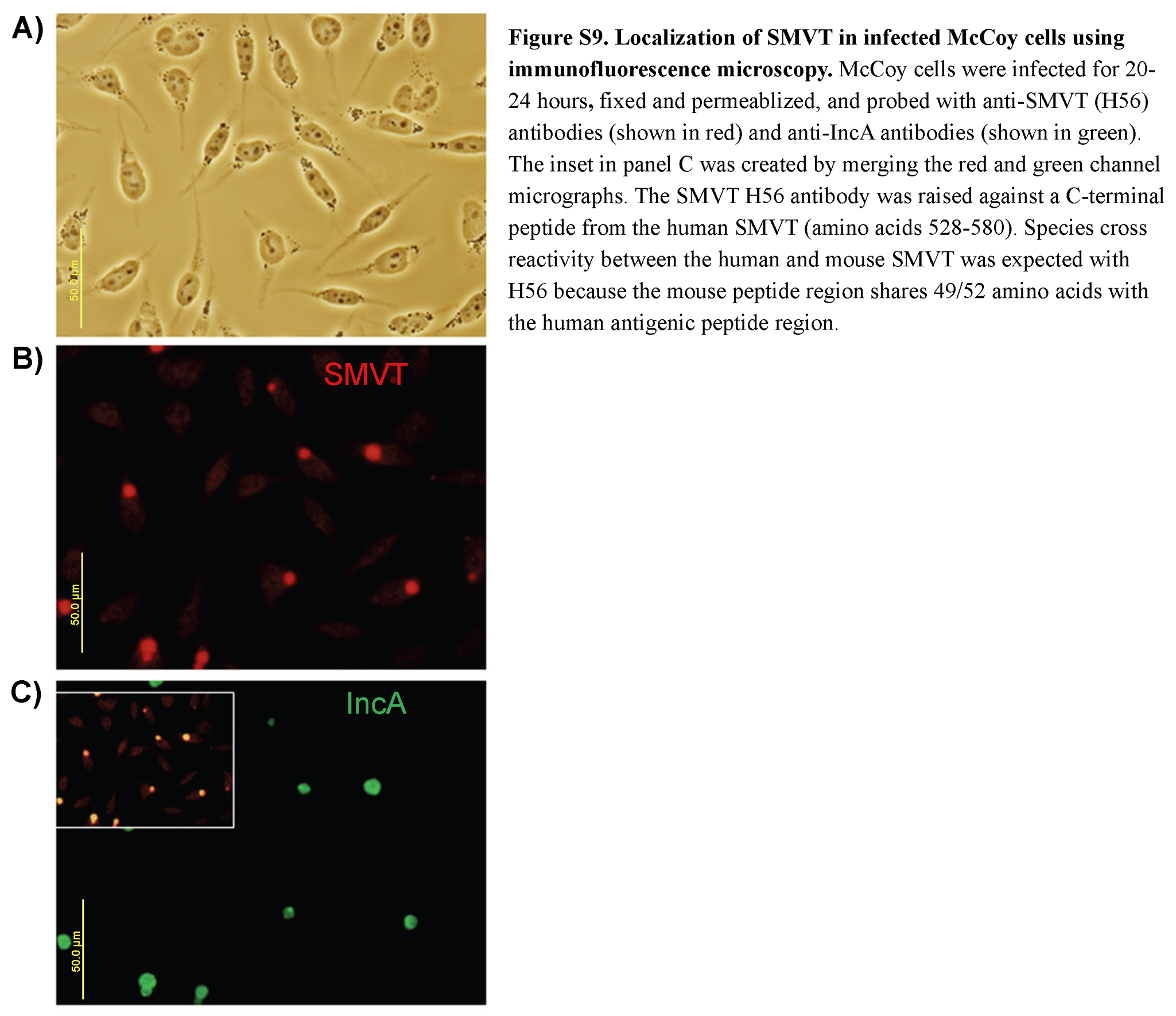

Supplement: Figure S9 — Localization of SMVT in infected McCoy cells using immunofluorescence microscopy. McCoy cells were infected for 20–24 hours, fixed and permeablized, and probed with anti-SMVT (H56) antibodies (shown in red) and anti-IncA antibodies (shown in green). The inset in panel C was created by merging the red and green channel micrographs. The SMVT H56 antibody was raised against a C-terminal peptide from the human SMVT (amino acids 528–580). Species cross reactivity between the human and mouse SMVT was expected with H56 because the mouse peptide region shares 49/52 amino acids with the human antigenic peptide region. (TIFF) [file pone.0046052.s009.tiff]
